# Generic versus brand-name drugs used in cardiovascular diseases

**DOI:** 10.1007/s10654-015-0104-8

**Published:** 2015-11-30

**Authors:** Lamberto Manzoli, Maria Elena Flacco, Stefania Boccia, Elvira D’Andrea, Nikola Panic, Carolina Marzuillo, Roberta Siliquini, Walter Ricciardi, Paolo Villari, John P. A. Ioannidis

**Affiliations:** Department of Medicine and Aging Sciences, University of Chieti, Via dei Vestini 5, 66013 Chieti, Italy; Regional Health Care Agency of Abruzzo, Via Attilio Monti 9, Pescara, Italy; Institute of Public Health, Catholic University of Rome, Largo Francesco Vito, 1, 00168 Rome, Italy; Department of Public Health and Infectious Diseases, Sapienza University of Rome, Viale Regina Elena 324, 00161 Rome, Italy; Department of Public Health Sciences, University of Turin, Via Santena 5bis, 10126 Turin, Italy; Italian National Institute of Health, Via Regina Elena 299, 00161 Rome, Italy; Stanford Prevention Research Center, Department of Medicine and Department of Health Research and Policy, Stanford University School of Medicine, Stanford, CA USA; Department of Statistics, Stanford University School of Humanities and Sciences, Stanford, CA USA; Meta-Research Innovation Center at Stanford (METRICS), Stanford, CA USA

**Keywords:** Generic drug, Brand-name drug, Cardiovascular diseases, Meta-analysis, Efficacy, Safety

## Abstract

This meta-analysis aimed to compare the efficacy and adverse events, either serious or mild/moderate, of all generic versus brand-name cardiovascular medicines. We searched randomized trials in MEDLINE, Scopus, EMBASE, Cochrane Controlled Clinical Trial Register, and ClinicalTrials.gov (last update December 1, 2014). Attempts were made to contact the investigators of all potentially eligible trials. Two investigators independently extracted and analyzed soft (including systolic blood pressure, LDL cholesterol, and others) and hard efficacy outcomes (including major cardiovascular adverse events and death), minor/moderate and serious adverse events. We included 74 randomized trials; 53 reported ≥1 efficacy outcome (overall sample 3051), 32 measured mild/moderate adverse events (n = 2407), and 51 evaluated serious adverse events (n = 2892). We included trials assessing ACE inhibitors (n = 12), anticoagulants (n = 5), antiplatelet agents (n = 17), beta-blockers (n = 11), calcium channel blockers (n = 7); diuretics (n = 13); statins (n = 6); and others (n = 3). For both soft and hard efficacy outcomes, 100 % of the trials showed non-significant differences between generic and brand-name drugs. The aggregate effect size was 0.01 (95 % CI −0.05; 0.08) for soft outcomes; −0.06 (−0.71; 0.59) for hard outcomes. All but two trials showed non-significant differences in mild/moderate adverse events, and aggregate effect size was 0.07 (−0.06; 0.20). Comparable results were observed for each drug class and in each stratified meta-analysis. Overall, 8 serious possibly drug-related adverse events were reported: 5/2074 subjects on generics; 3/2076 subjects on brand-name drugs (OR 1.69; 95 % CI 0.40–7.20). This meta-analysis strengthens the evidence for clinical equivalence between brand-name and generic cardiovascular drugs. Physicians could be reassured about prescribing generic cardiovascular drugs, and health care organization about endorsing their wider use.

**Electronic supplementary material:**

The online version of this article (doi:10.1007/s10654-015-0104-8) contains supplementary material, which is available to authorized users.

## Introduction

Because generic medicines are produced by multiple manufacturers after the patent of the brand-name equivalent expires, most of them are significantly less expensive than their brand-name counterparts [1]. To control pharmaceutical expenses, in the last two decades many payers and providers have encouraged the use of generic drugs, whose market share sharply increased and exceeded 40 % of the market volume in most developed countries in 2011 [2].

Generic drugs contain the same active ingredients as the original brand-name formulations and they aim to be identical to them or within an acceptable bioequivalent range in terms of their pharmacokinetic and pharmacodynamic characteristics. By extension, generics are considered identical in dose, strength, route of administration, safety, efficacy, and intended use [3].

Besides bioequivalence, the crucial assumption of identical health benefits of generics and brand-name drugs is based upon a few systematic reviews [1, 4–7]. In particular, in the context of cardiovascular diseases, which represents the first cause of disease burden in EU, USA and globally [8], only two meta-analyses directly compared the health outcomes of generic and brand-name medicines [4, 7]. One meta-analysis focused on clopidogrel only [7]. The other meta-analysis considered all drugs and included data on 30 randomized controlled trials (RCTs) [4]. However, it was published in 2008, and the results of dozens of RCTs reporting at least one efficacy or safety outcomes have been made available since then. Also, only efficacy outcomes from randomized trials had been considered in the prior meta-analysis, and the comparisons between generic and brand-name medicines lacked statistical power for several drug classes. For example, the conclusions on antiplatelet agents, ACE inhibitors and statins were based upon only 50, 23 and 71 subjects, respectively [4]. Finally, 16 of the 30 included trials had follow-up shorter than 48 h, a duration that allows bioequivalence measurements but provides very little information for safety or efficacy outcomes.

We updated and expanded previous work and carried out a meta-analysis of RCTs comparing the efficacy and adverse events, either serious or mild/moderate, of all generic versus brand-name cardiovascular drugs.

## Methods

### Search, study inclusion criteria and quality assessment

We included RCTs directly comparing at least one brand-name drug and at least one of its generic versions, and reporting at least one efficacy or safety outcome to treat or control cardiovascular diseases in humans, including vital signs (e.g. blood pressure), laboratory parameters used in clinical practice (e.g. low-density lipoprotein), mortality, and indices of morbidity. Trials focusing and reporting only on bioequivalence measures (e.g. drug serum concentration, time until maximum concentration, area under the curve based on serum concentration as a function of time) were retrieved to search whether safety and/or efficacy outcomes were also reported or could be retrieved from their investigators. Trials on biologic products (any medicinal product manufactured in or extracted from biological sources; e.g. vaccines) were excluded, as well as observational studies. No language or date restrictions were used.

The search was initially made online in MEDLINE, Scopus, EMBASE, Cochrane Controlled Clinical Trial Register (CCCTR), and ClinicalTrials.gov (last update December 1, 2014). The bibliographies of all relevant articles including reviews were then reviewed for further references. When it was not possible to extract any safety or efficacy outcome from a study, several attempts to contact the corresponding author were made. We planned to include also results that were posted in ClinicalTrials.gov and not published in peer-review journals. The search string was adjusted for each database while maintaining a common overall architecture. We used various combinations of the following terms related to four main domains: “generic* OR non-proprietary OR nonproprietary OR non-brand name drug” (title/abstract) AND “brand-name drug OR innovator OR patent drug OR proprietary drug” (all fields) AND “cardiovascular disease* OR coronary heart disease OR myocardial infarction OR acute coronary syndrome OR heart failure OR congestive heart disease OR blood pressure OR cholesterol OR hypertension OR hypercholesterolemia OR atherosclerosis OR antihypertensive* OR antiarrhythmic* OR beta blocker* OR calcium channel blocker* OR ace inhibitor* OR angiotensin receptor blocker* OR diuretic* OR statin*” (title/abstract) AND “random*” (all fields). Several alternative strings were used in PubMed by two investigators, independently, in order to enhance the sensitivity. Details on the adopted search strings are available in Additional Appendix S1. Although we could not exactly reproduce the search by Kesselheim et al. [4], as not enough details were available, we used the same main subject heading domains and, as a proof of principle for the sensitivity of our searches, we retrieved all 35 trials [9–46] found in the previous meta-analysis [4]. Indeed, the only substantial difference between the two searches were the online databases: we searched Scopus, CCCTR, and ClinicalTrials.gov in addition to MEDLINE and EMBASE.

We assessed aspects of the reported methodological quality of each study using the Cochrane risk of bias tool: generation of allocation sequences, allocation concealment, blinding, type of analysis, missing or selective outcome reporting and other sources of bias [47].

### Outcomes and data analysis

A standardized effect size (Cohen’s d) and its 95 % confidence intervals (95 % CIs) were computed for each study using the methodology detailed elsewhere [48–50]. In brief, the standardized effect sizes compare the difference in effect between the study groups divided by the standard deviation of this difference. This measure is independent of the measurement used, sample size, and standard deviation of the outcome measure, and allows the aggregation of different outcomes across studies to obtain effect sizes for each cardiovascular drug class as well as an aggregate effect size for all studies included in the meta-analysis [4, 51]. If the repeated measures correlation of a cross-over trial was not reported, we assumed a coefficient of 0.5 [4]. For continuous outcomes, if the standard deviation of the mean difference between the pre-post differences of the groups under comparison were not reported, we used the largest standard deviation of the mean differences of the groups [52]. For categorical outcomes, the natural logarithms of single study odds ratios were first computed and then divided by 1.81 to obtain the equivalent d [50]. If the sample size at the end of follow-up was not clearly specified, we extracted the baseline sample. An effect size lower than 0.2 should be considered very small; small if ranging from 0.2 to 0.5; medium from 0.5 to 0.8; and large if greater than 0.8 [51]. In our analysis, a positive effect size favors generics.

Two investigators independently extracted all outcomes. For efficacy outcomes, we initially selected for extraction the outcomes that were most closely related to the supposed clinical effect of the drug. For ACE inhibitors or Angiotensin receptor blockers, beta-blockers, calcium-channel blockers, and other drugs, we used systolic blood pressure reduction from baseline to the end of follow-up. For statins, we used LDL cholesterol reduction. For anticoagulants, we used the percentage of subjects within therapeutic INR range. For antiplatelet agents, we used bleeding time, or the variation in platelet aggregation inhibition, when bleeding time was not reported. For diuretics, we used the variation in urinary sodium excretion, or variation in urine volume, when urinary sodium secretion was not available, or systolic blood pressure, when both the above outcomes were not available. If the first choice outcomes were not available, we tried to contact authors for more information. In case of no response (as for all attempts), we extracted the other efficacy outcome among those listed above. In any case, all reported outcomes of any study were extracted, and the authors of all studies were contacted to request additional information of outcomes and on published or unpublished trials. Also, we requested information from the authors of the potentially eligible trials that were excluded because only bioequivalence outcome were reported.

For safety outcomes, we recorded separately mild/moderate and serious adverse events (any medical occurrence that resulted in death, life-threatening medical conditions, persistent or substantial disability or incapacity, or admission to hospital). When information was available, we only extracted the adverse events that were possibly related to the drug (as defined by the authors).

Hard efficacy outcomes (e.g. major cardiovascular events—MACE—or death) were extracted and combined separately from soft outcomes (e.g. systolic blood pressure or LDL reduction). Efficacy outcomes were reported at more than one follow-up point in several trials. We always extracted the data referred to the longest follow-up, except for five trials [33, 35, 40, 53, 54]. In these trials, the drug was administered only once, and efficacy data were reported at several time-points, some of which were distant from the estimated duration of the effect (based upon the half-life reported in study); we extracted the data referred to the time-point that was closer to the estimated duration of the drug effect (although, in all cases, the results of the various time-points did not vary substantially). Four other trials reported efficacy and safety data at different time points [25, 30, 34, 55]: we accordingly reported different follow-up durations for the same study in the meta-regression analyses on efficacy and safety outcomes. Finally, one trial compared two doses (25 and 50 mg) of generic and brand-name captopril [56]: the results were similar with both doses, and we extracted the data relative to the 50 mg dose. The details of the outcome extraction for each included study are reported in Additional Table S1.

The effect sizes of efficacy outcomes and mild/moderate adverse events were combined using a random-effect, generic inverse variance approach [47], and statistical heterogeneity was quantified using the I^2^ metric [57]. In case of limited data I^2^ metric 95 % confidence intervals are typically large [58] and thus inferences on the magnitude of the statistical heterogeneity should be cautious. Data on serious adverse events were combined using individual data random-effect logistic regression, with single study as the cluster unit, to avoid the exclusion of the many trials with zero events in both groups [47, 59]. The combined datasets with individual data were reconstructed using published 2X2 tables. The protocol of the review is available online as supporting information.

Several stratified meta-analyses were made to explore the potential influence of several a priori selected variables (health status: healthy, if defined so by the authors or without any major disease, or non-healthy; sample size: ≤30, 31–99, ≥100; study location: USA, Europe, Asia or other; design: cross-over or classic parallel group; follow-up duration: ≤1, 2–27, ≥28 days; blinding: open-label or single-blinded, double-blinded; funding: generic manufacturer, brand-name manufacturer, other funding, not reported). In addition to stratification, we used meta-regression with multiple covariates to explore potential predictors of the summary estimate of risk [60]. To reduce potential overfitting and false positive results, the number of variables included in both final and intermediate models (during modelling) was limited to 1 tenth of the included studies [57].

The impact of potential publication bias could not be evaluated as trials in this context are typically aimed at producing non-significant rather than significant results and indeed studies rarely had statistically significant differences in outcomes of interest. Standardized effect sizes and 95 % CIs for continuous outcomes were computed and combined using RevMan 5.3 (Copenhagen: The Nordic Cochrane Centre, The Cochrane Collaboration, 2014). Stata version 13 (Stata Corp., College Station, TX, 2013) was used to perform meta-regression and logistic regression analysis.

## Results

### Characteristics of eligible studies

Of the 745 papers initially retrieved (online PRISMA flow diagram), we included 74 randomized trials comparing generic vs brand-name drugs against cardiovascular diseases [9–13, 15–40, 42, 43, 53–56, 61–96]: 53 trials evaluated at least one efficacy outcome (overall sample 3051), 32 trials measured mild or moderate adverse events (n = 2407), and 52 reported on serious adverse events (n = 2952). Among the 53 trials including at least one efficacy outcome, we could extract hard outcomes (MACE or death) from 3 trials only, and soft outcomes from 52 trials. The safety outcomes of four trials were not published in peer-reviewed journal but were posted on ClinicalTrials.gov [61, 62, 65, 66]. 38 reports were excluded because only bioequivalence outcomes were reported or relevant outcome data could not be extracted. We attempted to contact all investigators, and thanks to their answers we were able to retrieve four additional trials [72, 73, 78, 81], and to add the data on mild/moderate [83] or serious [75] adverse events for two studies. The complete list of the excluded trials is available in Additional Appendix S2.

The main characteristics of each included trial have been reported in Table 1: the drug-classes under evaluation were ACE inhibitors or Angiotensin receptor blockers (12 trials), anticoagulants (n = 5), antiplatelet agents (n = 17), beta-blockers (n = 11), calcium channel blockers (n = 7); diuretics (n = 13); statins (n = 6); and others including alpha-blockers (n = 1); heparin (n = 1), and ezetimibe (n = 1).

**Table 1:** Characteristics of the 74 included randomized controlled trials

First author	Year	Country	Active principle	Patient’s status	Mean age (years)	Follow-up duration^a^	Design	Total sample (generics)	Extracted outcomes	Funding	Protocol registration
*ACE inhibitors or ARBs*											
Portoles [33]	2004	Spain	Enalapril	Healthy	23	5 h (36 h)	Crossover	23 (23)	SBP (E), mAEs (S), sAEs (S)	Not reported	No
Carranza [56]	2005	Mexico	Captopril	Hypertension	58	24 h	Cross-over	21 (21)	SBP (E)	Not reported	No
Kim (A) [64]	2009	Korea	Ramipril	Hypertension	50	8 weeks	Parallel-group	89 (45)	SBP (E), mAEs (S), sAEs (S)	Generic manufacturer	No
Spinola [69]	2009	Canada	Valsartan	Healthy	37	36 h	Cross-over	41 (41)	mAEs (S), sAEs (S)	Generic manufacturer	No
Iqbal [63]	2010	India	Valsartan	Healthy	25	24 h	Cross-over	18 (18)	sAEs (S)	Generic manufacturer	No
Jia [54]	2010	China	Losartan	Healthy	24	24 h (36 h)	Cross-over	27 (27)	SBP (E), mAEs (S), sAEs (S)	Generic manufacturer	No
Li [67]	2010	China	Olmesartan	Healthy	21	48 h	Cross-over	21 (21)	sAEs (S)	Generic manufacturer	2005L01077
Larouche 1 [65]^b^	2010	USA	Losartan	Healthy	42	2 weeks	Cross-over	80 (80)	mAEs (S), sAEs (S)	Generic manufacturer	NCT01124162
Larouche 2 [66]^b^	2010	USA	Losartan	Healthy	45	2 weeks	Cross-over	80 (80)	mAEs (S), sAEs (S)	Generic manufacturer	NCT01124175
Carlson 1 [61]^b^	2010	USA	Losartan and HCT	Healthy	42	2 weeks	Cross-over	80 (80)	sAEs (S)	Generic manufacturer	NCT01149473
Carlson 2 [62]^b^	2010	USA	Losartan and HCT	Healthy	42	2 weeks	Cross-over	20 (20)	mAEs (S), sAEs (S)	Generic manufacturer	NCT01149486
Oigman [68]	2013	Brazil	Ramipril and HCT	Hypertension	57	8 weeks	Parallel-group	102 (54)	SBP (E), mAEs (S), sAEs (S)	Generic manufacturer	ISRCTN 05051235
*Anticoagulants*											
Handler [19]	1998	USA	Warfarin	Atrial fibrillation	71	4 weeks	Cross-over	55 (54)	% within INR range (E), Anticoagulation events (S), sAEs (S)	Generic manufacturer	No
Neutel [28]	1998	USA	Warfarin	Atrial fibrillation	70	5 weeks	Cross-over	39 (39)	% within INR range (E)	Not reported	No
Weibert [42]	2000	USA	Warfarin	Atrial fibrillation	70	4 weeks	Cross-over	104 (102)	mAEs (S), sAEs (S)	Generic manufacturer	No
Lee [22]	2005	Taiwan	Warfarin	Heart Valves	52	12 weeks	Cross-over	35 (35)	% within INR range (E) Anticoagulation events (S), sAEs (S)	Industry (other)	No
Pereira [31]	2005	Canada	Warfarin	Outpatients to be treated	63	15 weeks	Cross-over	7 (7)	% within INR range (E)	Not reported	No
*Antiplatelet agents*											
Rao [34]	2003	India	Clopidogrel	Healthy	27	2 h (10 days)	Parallel-group	20 (10)	Bleeding time (E), mAEs (S), sAEs (S)	Generic manufacturer	No
Ashraf [9]	2005	Pakistan	Clopidogrel	CVD	49	24 h	Cross-over	30 (30)	Platelet aggr. inhibition (E), sAEs (S)	Non profit	No
Mijares [73]	2008	Venezuela	Clopidogrel	Healthy	30	2 weeks	Cross-over	20 (20)	Platelet aggr. inhibition (E), mAEs (S), sAEs (S)	Industry (other)	No
Kim (P) [55]	2009	Korea	Clopidogrel	Healthy	24	7 days (13 days)	Cross-over	44 (44)	Platelet aggr. inhibition (E), mAEs (S), sAEs (S)	Generic manufacturer	No
Di Girolamo [70]	2010	Argentina	Clopidogrel	Healthy	34	12 h	Cross-over	24 (24)	sAEs (S)	Generic manufacturer	No
Müller [74]	2010	Venezuela	Clopidogrel	Healthy	23	7 days	Cross-over	20 (20)	Platelet aggr. inhibition (E)	Not reported	No
Shim [95]	2010	Korea	Clopidogrel	Healthy	29	1 week	Cross-over	29 (29)	Bleeding time (E), sAEs (S)	Generic manufacturer	No
Khosravi [71]	2011	Iran	Clopidogrel	PCI	59	6 months	Parallel-group	442 (224)	MACE and death (E)	Generic manufacturer	IRCT 138712111723N1
Suh [96]	2011	Korea	Clopidogrel	CVD	62	4 weeks	Parallel-group	203 (100)	mAEs (S), sAEs (S)	Generic manufacturer	NCT00947843
Oberhänsli [75]	2012	Swiss	Clopidogrel	CVD	69	10 days	Cross-over	60 (60)	Platelet aggr. inhibition (E), sAEs (S)	Non profit	No
Srimahachota [78]	2012	Thailand	Clopidogrel	CVD	NR	6 h	Parallel-group	49 (25)	Platelet aggr. inhibition (E), mAEs (S)	Not reported	No
Tsoumani (A) [79]	2012	Greece	Clopidogrel	ACS	70	6 months	Parallel-group	86 (45)	Platelet aggr. inhibition (E)	Generic manufacturer	No
Tsoumani (E) [80]	2012	Greece	Clopidogrel	ACS	64	4 weeks	Parallel-group	96 (51)	Platelet aggr. inhibition (E)	Non profit	No
Zou [81]	2012	China	Clopidogrel	Healthy	24	36 h	Cross-over	20 (20)	sAEs (S)	Not reported	No
Park (J) [76]	2013	Korea	Clopidogrel	CVD	62	4 weeks	Parallel-group	130 (65)	Platelet aggr. inhibition (E), MACE and death (E), mAEs (S), sAEs (S)	Generic manufacturer	NCT01584791
Komosa [72]	2014	Poland	Clopidogrel	CVD	49	8 days	Parallel-group	53 (28)	Platelet aggr. inhibition (E), mAEs (S)	Not reported	No
Seo [77]	2014	Korea	Clopidogrel	ACS	58	24 h (4 weeks for AEs and MACE)	Parallel-group	95 (47)	Platelet aggr. inhibition (E), MACE and death (E), sAEs (S)	Generic manufacturer	NCT02060786
*Beta*-*blockers*											
Biswas [82]	1989	India	Propranolol	Healthy	26	7 days	Cross-over	18 (18)	SBP (E)	Not reported	No
Carter [13]	1989	USA	Propranolol	Hypertension	46	4 weeks	Cross-over	12 (12)	SBP (E)	Non profit	No
el-Sayed [16]	1989	UK	Propranolol	Healthy	20	2 h	Cross-over	12 (12)	SBP (E)	Not reported	No
Chiang [15]	1995	Taiwan	Atenolol	Hypertension	59	4 weeks	Cross-over	23 (23)	SBP (E)	Not reported	No
Sarkar [35]	1995	USA	Atenolol	Healthy	NR	12 h (24 h)	Cross-over	29 (29)	SBP (E), sAEs (S)	Generic manufacturer	No
Bongers [12]	1999	Germany	Metoprolol	Stable angina	62	4 weeks	Cross-over	51 (51)	SBP (E), mAEs (S), sAEs (S)	Brand-name manufacturer	No
Cuadrado [53]	2002	Spain	Atenolol	Healthy	23	24 h (30 h)	Cross-over	24 (24)	SBP (E), mAEs (S), sAEs (S)	Generic manufacturer	No
Mirfazaelian [26]	2003	Iran	Atenolol	Healthy	36	24 h	Cross-over	12 (12)	SBP (E), sAEs (S)	Not reported	No
Portoles [32]	2005	Spain	Carvedilol	Healthy	23	24 h	Cross-over	24 (24)	sAEs (S)	Not reported	No
Bus-Kwasnik [83]	2012	Poland	Bisoprolol	Healthy	23	60 h	Cross-over	24 (24)	mAEs (S), sAEs (S)	Generic manufacturer	EudraCT 2009 014861-20
Liu [84]	2013	China	Carvedilol	Healthy	27	24 h	Cross-over	23 (23)	mAEs (S), sAEs (S)	Generic manufacturer	No
*Calcium channel blockers*											
Usha [40]	1997	India	Diltiazem	Healthy	27	12 h	Cross-over	12 (12)	SBP (E)	Generic manufacturer	No
Saseen [36]	1997	USA	Verapamil	Hypertension	70	2 weeks	Cross-over	8 (8)	SBP (E), sAEs (S)	Not reported	No
Park [30]	2004	Korea	Amlodipine	Healthy	22	7 h (6 days)	Cross-over	18 (18)	SBP (E), sAEs (S)	Not reported	No
Kim [21]	2007	Korea	Amlodipine	Hypertension	53	8 weeks	Parallel-group	188 (94)	SBP (E), mAEs (S), sAEs (S)	Generic manufacturer	No
Mignini [25]	2007	Italy	Amlodipine	Healthy	35	3 h (6 days)	Cross-over	24 (24)	SBP (E), mAEs (S), sAEs (S)	Not reported	No
Kim [85]	2008	Korea	Amlodipine	Hypertension	53	8 weeks	Parallel-group	124 (63)	SBP (E), mAEs (S), sAEs (S)	Generic manufacturer	No
Liu [86]	2009	China	Amlodipine	Healthy	21	5 days	Cross-over	20 (20)	mAEs (S), sAEs (S)	Non profit	No
*Diuretics*											
Garg [17]	1984	India	Furosemide	Healthy	33	6 h	Cross-over	16 (16)	SBP (E), mAEs (S), sAEs (S)	Not reported	No
Grahnen [18]	1984	Sweden	Furosemide	Healthy	26	7 h	Cross-over	8 (8)	Urine volume (E)	Not reported	No
Martin [23]	1984	UK	Furosemide	Healthy	30	24 h	Cross-over	12 (12)	Urine sodium (E)	Non profit	No
Pan [29]	1984	Hong Kong	Furosemide	CVD	NR	8 h	Cross-over	5 (5)	Urine sodium (E)	Not reported	No
Meyer [24]	1985	S. Africa	Furosemide	Healthy	29	6 h	Cross-over	12 (12)	Urine volume (E)	Not reported	No
Singh [38]	1987	India	Furosemide	Edema of renal origin	36	6 h	Cross-over	7 (7)	SBP (E), sAEs (S)	Not reported	No
Sharoky [37]	1989	USA	HCT and Triamterene	Hypertension	55	3 weeks	Cross-over	30 (30)	SBP (E), sAEs (S)	Generic manufacturer	No
Kaojarern [20]	1990	Thailand	Furosemide	Healthy	32	8 h	Cross-over	8 (8)	Urine sodium (E)	Brand-name manufacturer	No
Awad [11]	1992	Jordan	Furosemide	Healthy	27	8 h	Cross-over	20 (20)	Urine sodium (E)	Not reported	No
Murray [27]	1997	USA	Furosemide	CVD	65	2 weeks	Cross-over	17 (17)	Urine sodium (E)	Brand-name manufacturer	No
Almeida [87]	2011	Portugal	Eplerenone	Healthy	40	24 h	Cross-over	27 (27)	mAEs (S), sAEs (S)	Generic manufacturer	No
Kumar (India) [88]	2014	India	Losartan and HCT	Healthy	33	48 h	Cross-over	15 (15)	sAEs (S)	Generic manufacturer	No
Kumar (Japan) [88]	2014	Japan	Losartan and HCT	Healthy	30	48 h	Cross-over	24 (24)	sAEs (S)	Generic manufacturer	No
*Statins*											
Assawawitoontip [10]	2002	Thailand	Simvastatin	Hypercholest.	37	8 weeks	Cross-over	48 (48)	LDL (E)	Generic Manufacturer	No
Wiwanitkit [43]	2002	Thailand	Simvastatin	Healthy	49	16 weeks	Cross-over	37 (37)	LDL (E), sAEs (S)	Generic manufacturer	No
Kim [91]	2010	Korea	Atorvastatin	CVD	61	8 weeks	Parallel-group	235 (119)	LDL (E), mAEs (S), sAEs (S)	Generic manufacturer	NCT01029522.
Liu [93]	2010	China	Atorvastatin	Healthy	24	48 h	Cross-over	45 (45)	sAEs (S)	Brand-name manufacturer	CNR 2007L02512
Boh [89]	2011	Slovenia	Atorvastatin	Hypercholest.	56	16 weeks	Parallel-group	137 (66)	LDL (E), mAEs (S), sAEs (S)	Not reported	No
Kim [92]	2013	Korea	Atorvastatin	Hypercholest.	61	8 weeks	Parallel-group	289 (143)	LDL (E), mAEs (S), sAEs (S)	Generic manufacturer	NCT01285544.
*Others*											
Tsai [39]	2007	Taiwan	Terazosin	Healthy	64	6 weeks	Cross-over	43 (43)	SBP (E), mAEs (S), sAEs (S)	Generic manufacturer	No
Feng [90]	2009	China	Heparin	Healthy	21	24 h	Cross-over	22 (22)	sAEs (S)	Not reported	No
Palmer [94]	2014	India	Ezetimibe	Healthy	27	72 h	Cross-over	51 (50)	sAEs (S)	Generic manufacturer	NCT01597700

*NR* not reported, *HCT* hydrochlorothiazide, *ACE* angiotensin-converting-enzyme, *ARBs* angiotensin II receptor blockers, *SBP* systolic blood pressure, *LDL* low-density lipoprotein, *sAEs* serious adverse events, *mAEs* mild adverse events, *Hypercholest.* hypercholesterolemia, *CVD* cardiovascular diseases, *ACS* acute coronary syndrome, *PCI* percutaneous coronary intervention, (*E*) efficacy outcome, (*S*) safety outcome, *MACE* major cardiovascular events, *INR* international normalized ratio

^a^When the follow-up duration differed between safety and efficacy outcomes, the follow-up of the safety outcome has been reported under brackets (see text and Additional Table S1 for details)

^b^Results posted in ClinicalTrials.gov only

Of the 74 trials, 39 trials were performed in Asian countries, 15 in Europe and 18 in America; 24 studies had a follow-up duration equal or longer than 4 weeks; 58 trials had a cross-over design; the sample size was ≥100 in 10 trials, while 40 studies included 30 subjects or less; 37 trials were funded by the generic manufacturer, and only 11 of the 37 studies published after 2005 had the protocol registered online (11/27 from 2010, the year in which the first trial with a registered protocol was published).

All outcomes evaluated in each trial are listed in Additional Table S1: the extracted outcomes varied across single studies, however an outcome that was closely related to the supposed clinical effect of the drug was extracted in all trials with at least one efficacy outcome, with two exceptions that were excluded [14, 41]. The mean difference between groups in systolic blood pressure change from baseline was extracted in 18 of the 18 trials with efficacy outcomes on beta-blockers, ACE inhibitors (or Angiotensin receptor blockers) and calcium channel blockers. Also, the variation in LDL cholesterol was extracted from all studies on statins.

As shown in Additional Table S2, based on their reporting 7 of the 70 included trials were at low risk of bias for at least 5 of the 6 methodological characteristics included in Cochrane risk-of-bias assessment tool, while 14 “scored” 1 or 0. As regards the single items, the random sequence generation and allocation concealment were unclear or inappropriate for 35 and 60 studies, respectively. Only 24 trials were double-blinded, and 27 had low risk of selective reporting.

### Efficacy

**Fig. 1:**
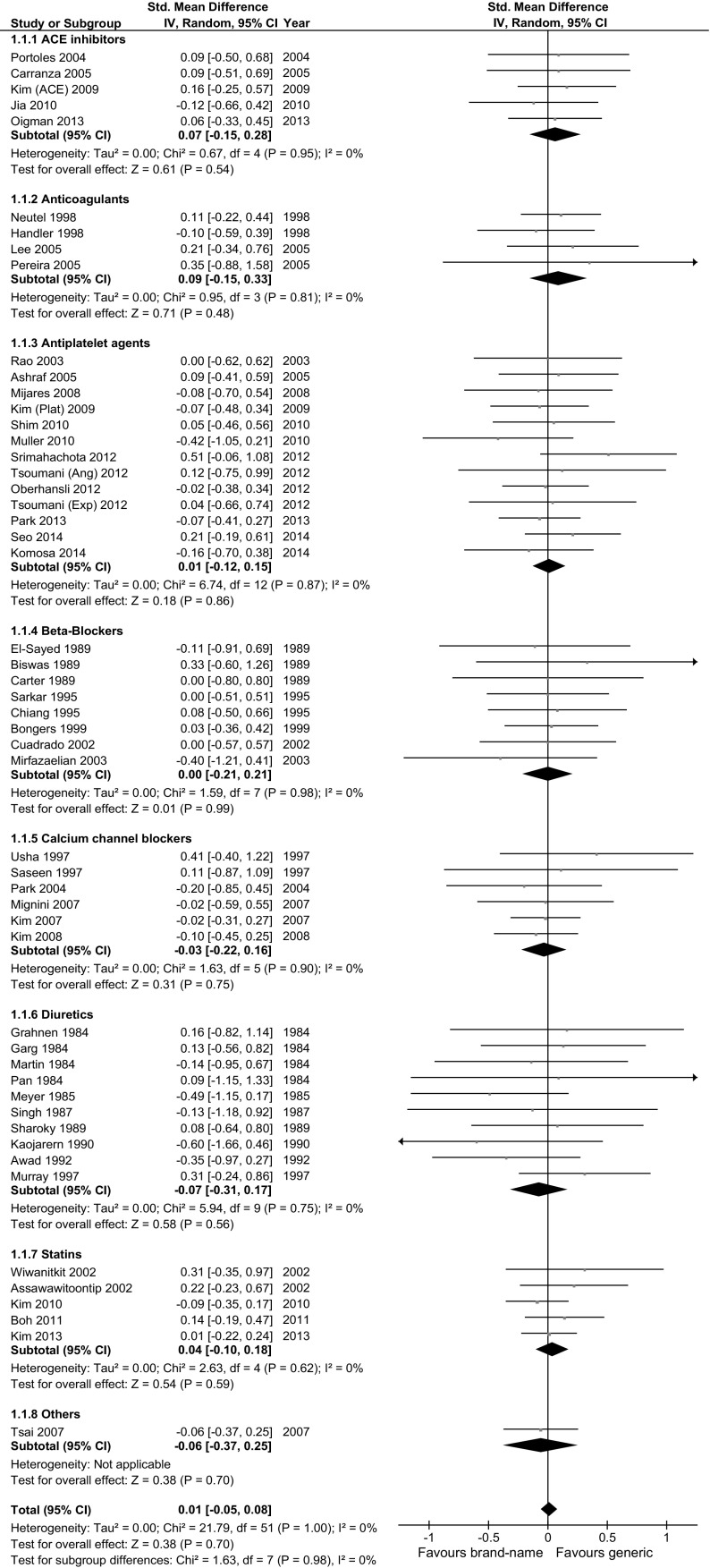
Meta-analysis evaluating the clinical efficacy (soft outcomes) of generic vs brand-name drugs against cardiovascular diseases

**Fig. 2:**
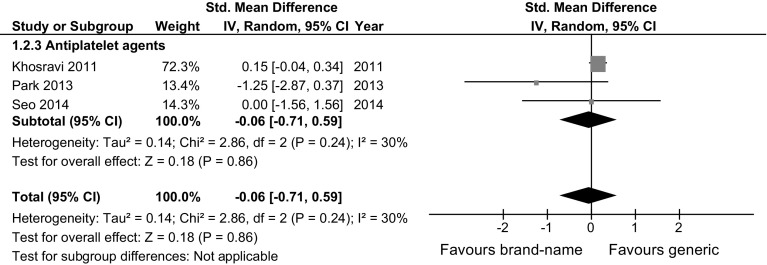
Meta-analysis evaluating the clinical efficacy (hard outcomes: MACE or death) of generic versus brand-name drugs against cardiovascular diseases

Overall, 52 trials including 2609 subjects were included in the meta-analysis evaluating soft efficacy outcomes (Table 1; Fig. 1), and 3 trials including 667 subjects were included in the meta-analysis evaluating hard efficacy outcomes (Fig. 2). For both soft and hard outcomes, all RCTs (100 %) showed non-significant differences between generic and brand-name drugs. The aggregate effect size was 0.01 (95 % CI −0.05; 0.08) for soft outcomes; −0.06 (95 % CI −0.71; 0.59) for hard outcomes, both indicating no difference between generic and brand-name drugs. Similar results were observed for each drug class and in each stratified meta-analysis (Table 2). There was no large statistical heterogeneity between studies in any of the comparisons. No covariate was significantly associated with effect size in meta-regression analysis (Additional Table S3).

**Table 2:** Results of meta-analyses comparing the efficacy and safety of generic versus brand-name drugs against cardiovascular diseases

	Efficacy (soft outcomes)		I^2^ (%)	Mild or moderate adverse events		I^2^ (%)
	n (N)	ES (95 % CI)	Upper (95 % CI)	n (N)	ES (95 % CI)	Upper (95 % CI)
Overall	52 (2609)	0.01 (−0.05; 0.08)	0 (32)	32 (2407)	0.07 (−0.06; 0.20)	15 (45)
*Drug class*						
ACE inhibitors or ARBs	5 (262)	0.07 (−0.15; 0.28)	0 (79)	8 (456)	0.10 (−0.20; 0.41)	38 (72)
Anticoagulants	4 (136)	0.09 (−0.15; 0.33)	0 (85)	3 (196)	0.19 (−0.12; 0.50)	0 (90)
Antiplatelet agents	13 (732)	0.01 (−0.12; 0.15)	0 (57)	7 (519)	−0.10 (−0.40; 0.19)	0 (71)
Beta-blockers	8 (181)	0.00 (−0.21; 0.21)	0 (68)	4 (123)	0.23 (−0.10; 0.57)	0 (85)
Calcium channel blockers	6 (374)	−0.03 (−0.22; 0.16)	0 (75)	4 (356)	0.09 (−0.23; 0.42)	0 (85)
Diuretics	10 (135)	−0.07 (−0.31; 0.17)	0 (62)	2 (43)	0.27 (−1.81; 2.36)	91 (–)
Statins	5 (746)	0.04 (−0.10; 0.18)	0 (79)	3 (671)	−0.06 (−0.40; 0.27)	16 (91)
Others	1 (43)	−0.06 (−0.37; 0.25)	–	1 (43)	0.05 (−0.43; 0.53)	–
*Health status*						
Healthy	23 (488)	−0.06 (−0.19; 0.06)	0 (45)	17 (557)	0.14 (−0.13; 0.40)	38 (65)
Non-healthy	29 (2121)	0.05 (−0.03; 0.13)	0 (41)	15 (1850)	0.05 (−0.09; 0.19)	0 (53)
*Continent*						
America	11 (258)	0.03 (−0.14; 0.21)	0 (60)	7 (402)	0.11 (−0.16; 0.37)	36 (73)
Europe	12 (586)	0.02 (−0.13; 0.17)	0 (60)	8 (365)	0.15 (−0.15; 0.46)	24 (65)
Asia	27 (1651)	0.01 (−0.07; 0.10)	0 (44)	16 (1545)	0.02 (−0.16; 0.21)	4 (52)
Others	2 (114)	−0.15 (−0.67; 0.37)	49 (–)	1 (95)	−0.22 (−0.67; 0.23)	–
Funding						
Industry—generic	20 (1721)	0.01 (−0.08; 0.09)	0 (47)	22 (2009)	0.05 (−0.08; 0.18)	10 (44)
Industry—brand-name	2 (25)	−0.03 (−0.89; 0.83)	55 (–)	0 (0)	–	–
Other funding	7 (321)	0.02 (−0.21; 0.24)	0 (71)	3 (75)	−0.35 (−0.99; 0.29)	0 (90)
Not reported	22 (552)	−0.01 (−0.12; 0.14)	0 (46)	7 (323)	0.48 (0.04; 0.92)	28 (69)
*Follow*-*up duration*						
≤1 day	22 (484)	0.00 (−0.13; 0.14)	0 (46)	4 (115)	0.23 (−0.72; 1.81)	73 (91)
2–27 days	10 (299)	−0.02 (−0.19; 0.16)	0 (62)	14 (501)	0.16 (0.09; 0.41)	10 (48)
≥28 days	20 (1826)	0.02 (−0.06; 0.11)	0 (48)	14 (1791)	0.04 (−0.09; 0.18)	0 (55)
*Study design*						
Parallel-group	14 (1693)	0.02 (−0.08; 0.12)	0 (55)	12 (1622)	−0.03 (−0.19; 0.14)	0 (58)
Cross-over	38 (916)	0.00 (−0.09; 0.10)	0 (37)	20 (805)	0.15 (−0.04; 0.34)	27 (58)
*Blinding*						
Open-label or single-blind	30 (1557)	0.01 (−0.07; 0.10)	0 (40)	25 (1452)	0.09 (−0.05; 0.23)	0 (44)
Double-blind	22 (1052)	0.02 (−0.09; 0.12)	0 (46)	7 (955)	0.07 (−0.22; 0.36)	52 (80)
*Sample size*						
≤30	28 (512)	−0.01 (−0.14; 0.12)	0 (42)	11 (249)	0.19 (−0.23; 0.60)	46 (73)
31–99	17 (892)	0.06 (−0.05; 0.17)	0 (51)	13 (738)	0.13 (−0.04; 0.30)	0 (57)
≥100	7 (1205)	−0.01 (−0.13; 0.10)	0 (71)	8 (1420)	−0.01 (−0.19; 0.18)	10 (71)

A positive effect size favors generics

*ES* effect size, *OR* odds ratio, *CI* confidence interval, *n* number of trials, (*N*) number of participants, *ARBs* angiotensin II receptor blockers

### Mild or moderate adverse events

**Fig. 3:**
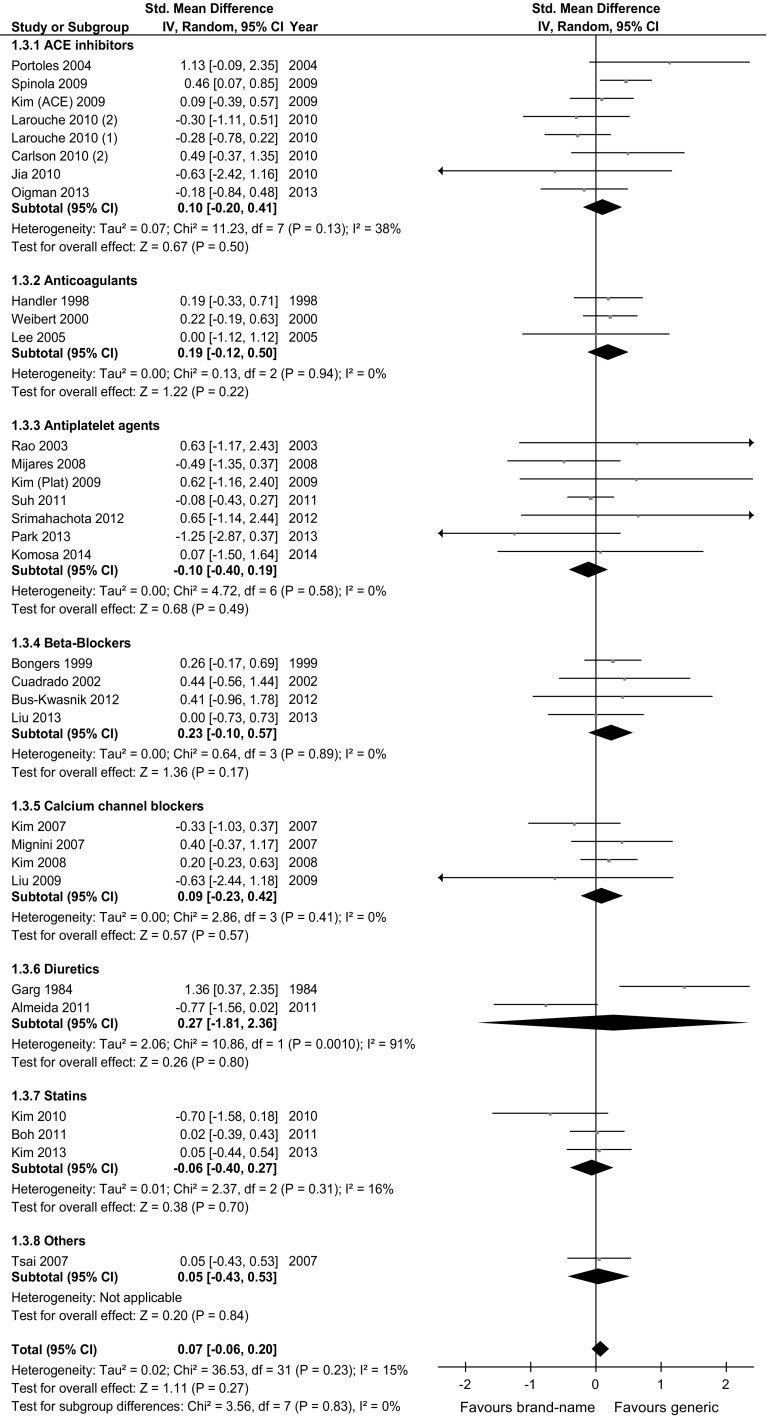
Meta-analysis evaluating the risk of mild or moderate adverse events of generic versus brand-name drugs against cardiovascular diseases

Overall, 32 trials including 2407 subjects were included in the meta-analysis evaluating mild or moderate adverse events. All but 2 trials showed non-significant differences between generic and brand-name drugs, and aggregate effect size was 0.07 (95 % CI −0.06; 0.20; Table 2; Fig. 3). Comparable results were observed for each drug class and in each stratified meta-analysis (Table 2). The statistical heterogeneity between studies was low or moderate in most comparisons. A significant difference in the risk of mild or moderate adverse events favoring generic versus brand-name drugs was found in two stratified meta-analyses (trials not reporting the sponsor; trials of intermediate follow-up duration). However, none of such covariates was significantly associated with effect size in meta-regression analysis, either univariate or multivariate (Additional Table S4).

### Serious adverse events

Overall, 8 serious possibly drug-related adverse events were reported in 52 studies: 5 events among the 2134 subjects who assumed generics (819 from parallel-group RCTs and 1315 from cross-over RCTs; rate = 2.34 ‰); 3 events among the 2136 subjects who assumed brand-name drugs (815 from parallel-group RCTs and 1321 from cross-over RCTs; rate = 1.40 ‰). All but 1 event were reported by industry-sponsored Asian studies, published after the year 2005, with follow-up longer than 27 days [22, 39, 92, 96]. No deaths were reported, and all cases made a full recovery. The meta-analysis on serious adverse events showed no significant differences between generic and brand-name medicines (odds ratio for generics: 1.69; 95 % CI 0.40; 7.20). No stratified analysis produced significant results.

## Discussion

Every clinician is repeatedly exposed to anecdotal evidence from patients, colleagues, and of course company representatives, claiming that generic drugs are not as effective and/or safe as their branded counterparts [97–99]. A number of observational studies [4, 5, 100, 101] show good results with generics, however their use is still modest in several countries [102, 103]. Brand name antiplatelet agents, ACE inhibitors, Angiotensin receptor blockers and statins have combined sales which exceed $100 billion yearly and dominate the cardiovascular pharmaceutical market [104–106]. The only other published meta-analysis to-date on this topic, although rigorously done, only included 50, 23 and 71 subjects in the evaluation of antiplatelet agents, ACE inhibitors and statins, respectively [4]. Also, most of these subjects had been followed for <2 days, only efficacy outcomes were combined, and many trials have been published later on [32, 54, 55, 63, 64, 67–72, 74–81, 83, 84, 86–96]. In the present meta-analysis all 53 RCTs evaluating efficacy outcomes found no significant differences between generic and brand-name cardiovascular drugs. Unsurprisingly, the combined estimate of efficacy did not approach significance for any drug-class and in any stratified analysis, all of which showed low or moderate between-study heterogeneity. Similarly, the risk of mild or moderate adverse events was comparable between generics and branded medicines in 26 out of 29 trials, and combining trial results no evidence emerged of a superiority of one drug type over the other. Concerning serious adverse events, the results of 47 studies showed again a similar risk with both generics and proprietary drugs, however the number of events was sparse, and further data must be collected to achieve satisfactory statistical power.

Taken together, these results suggest that using generic instead of brand-name cardiovascular drugs does not imply a loss in either efficacy or safety. These findings provide a more solid confirmation to observational analyses and to the previous meta-analysis on randomized trials, with respect to which we included from 10 to 15 times more subjects consuming statins, ACE inhibitors and antiplatelet agents, and included 24 versus 7 trials with a follow-up of 4 weeks or more [4].

Our study has some potential limitations that must be mentioned. First, approximately half of the included studies were bioequivalence trials with cross-over design, short follow-up duration and small samples, sometimes including disproportionately young and healthy participants. Although the validity of such trials to demonstrate differences in clinical outcomes may be debated, it is worth noting that similar results were obtained combining trials with a parallel-group design, larger samples, longer follow-up, and including only unhealthy subjects, and age was not associated with the effect estimate in meta-regression analysis. In any case, age, multimorbidity, polypharmacy and a greater degree of compromised metabolic function might have influenced the bioequivalence of generic drugs and, due to limited evidence from RCTs, the influence of these parameters cannot be studied. Second, most of the included studies did not report the funding source or were sponsored by the generic manufacturer, thus it is very difficult to draw any firm conclusions about the potential impact of any sponsorship bias, even if the stratified analyses and meta-regression revealed no clear patterns. Given that the vast majority of trials in this field have very low power to detect significant differences, it is unlikely that a lot of significant differences have been generated and then suppressed by sponsors. Third, we combined together different outcomes of efficacy, and adverse events that were heterogeneously measured. However, within several drug-classes (ACE inhibitors or Angiotensin receptor blockers, anticoagulants, beta-blockers, calcium-channel blockers and statins) the extracted efficacy outcomes were identical. Moreover, for the other drug-classes the alternative efficacy outcomes that could be extracted from single studies never showed a different result (Additional Table S1), and both soft and hard outcomes were always non-significant in single studies. Fourth, almost two-thirds of all studies were at high-risk of selective outcome reporting, less than one-third of the trials published after 2005 had their protocol registered online, and most cross-over studies failed to accommodate the within-individual differences in the analysis. Therefore, on one side there is the need of an in-depth analysis of the publication pattern of generic trials starting from clinical trial registries, as a relevant proportion of RCTs likely remained unpublished [107]. On the other side, more journals in the field should adhere to ICMJE recommendation of trial protocol registration in a public trials registry as a condition for publication [108]. Fifth, it must be noted that more studies on drugs with narrow therapeutic interval like antiarrhythmics are strongly needed, and that even though we found no differences between generics and brand-name drugs, there might be differences concerning adverse events between two generic drugs. Finally, the overall meta-analytic estimate combining the results of different drug classes is typically problematic and should be interpreted with caution, because for some drugs the influence of the preparation (inert binders, fillers, manufacturing process) might be bigger than for other drugs, even though the mechanism of the drug is the same for generic and brand name drugs. Unfortunately, we could not dissect the potential influence of the preparation as this information was generally not available across the included studies.

## Conclusions

The present meta-analysis confirmed the substantial clinical equivalence between brand-name and generic cardiovascular drugs. On one side, this finding is based upon a suboptimal evidence: very few studies used hard outcomes and followed patients for more than 3 months, and many trials were conducted in healthy volunteers. On the other side, the relatively large randomized sample size, the inclusion of many trials with a follow-up longer than 4 weeks, the stability of the results, and the inclusion of adverse events in the analyses may provide a more solid reassurance to the scientific community, possibly contributing to reduce the claims and mistrust towards generic medications [109]. Although generic prices also may substantially raise following opportunistic behaviors [110], the growing availability as generic products of blockbuster drugs is likely to produce several USD billions of savings [111, 112]. Physicians could be reassured about prescribing generic cardiovascular drugs to patients, and health care organizations about endorsing their larger use.

## Electronic supplementary material

Below is the link to the electronic supplementary material.

Supplementary material 1 (DOC 1190 kb)

Supplementary material 2 (DOC 60 kb)

## References

[1] Dentali F, Donadini MP, Clark N, Crowther MA, Garcia D, Hylek E (2011). Brand name versus generic warfarin: a systematic review of the literature. Pharmacotherapy.

[2] OECD (2013). OECD. Health at a glance 2013: OECD indicators.

[3] Food and Drug Administration. Generic drugs: questions and answers. US Food and Drug Administration. 2013. http://www.fda.gov/Drugs/ResourcesForYou/Consumers/QuestionsAnswers/ucm100100.htm. Accessed November 2014.

[4] Kesselheim AS, Misono AS, Lee JL, Stedman MR, Brookhart MA, Choudhry NK (2008). Clinical equivalence of generic and brand-name drugs used in cardiovascular disease: a systematic review and meta-analysis. JAMA.

[5] Kesselheim AS, Stedman MR, Bubrick EJ, Gagne JJ, Misono AS, Lee JL (2010). Seizure outcomes following the use of generic versus brand-name antiepileptic drugs: a systematic review and meta-analysis. Drugs..

[6] Talati R, Scholle JM, Phung OP, Baker EL, Baker WL, Ashaye A (2012). Efficacy and safety of innovator versus generic drugs in patients with epilepsy: a systematic review. Pharmacotherapy.

[7] Caldeira D, Fernandes RM, Costa J, David C, Sampaio C, Ferreira JJ (2013). Branded versus generic clopidogrel in cardiovascular diseases: a systematic review. J Cardiovasc Pharmacol.

[8] Murray CJ, Lopez AD (2013). Measuring the global burden of disease. N Engl J Med.

[9] Ashraf T, Ahmed M, Talpur MS, Kundi A, Faruqui AM, Jaffery AH (2005). Competency profile of locally manufactured clopidogrel Lowplat and foreign manufactured clopidogrel Plavix in patients of suspected ischemic heart disease (CLAP-IHD). J Pak Med Assoc.

[10] Assawawitoontip S, Wiwanitkit V (2002). A randomized crossover study to evaluate LDL-cholesterol lowering effect of a generic product of simvastatin (Unison Company) compared to simvastatin (Zocor) in hypercholesterolemic subjects. J Med Assoc Thai.

[11] Awad R, Arafat T, Saket M, Saleh M, Gharaibeh M, Zmeili S (1992). A bioequivalence study of two products of furosemide tablets. Int J Clin Pharmacol Ther Toxicol.

[12] Bongers V, Sabin GV (1999). Comparison of the effect of two metoprolol formulations on total ischaemic burden. Clin Drug Invest.

[13] Carter BL, Gersema LM, Williams GO, Schabold K (1989). Once-daily propranolol for hypertension: a comparison of regular-release, long-acting, and generic formulations. Pharmacotherapy.

[14] Carter BL, Noyes MA, Demmler RW (1993). Differences in serum concentrations of and responses to generic verapamil in the elderly. Pharmacotherapy.

[15] Chiang HT, Hou ZY, Lee DK, Wu TL, Chen CY (1995). A comparison of antihypertensive effects between two formulations of atenolol: tenolol and tenormin. Zhonghua Yi Xue Za Zhi (Taipei).

[16] El-Sayed MS, Davies B (1989). Effect of two formulations of a beta blocker on fibrinolytic response to maximum exercise. Med Sci Sports Exerc.

[17] Garg SK, Gupta U, Mathur VS (1984). Comparative bioequivalence study of furosemide in human volunteers. Int J Clin Pharmacol Ther Toxicol.

[18] Grahnen A, Hammarlund M, Lundqvist T (1984). Implications of intraindividual variability in bioavailability studies of furosemide. Eur J Clin Pharmacol.

[19] Handler J, Nguyen TT, Rush S, Pham NT (1998). A blinded, randomized, crossover study comparing the efficacy and safety of generic Warfarin sodium to Coumadin. Prev Cardiol..

[20] Kaojarern S, Poobrasert O, Utiswannakul A, Kositchaiwat U (1990). Bioavailability and pharmacokinetics of furosemide marketed in Thailand. J Med Assoc Thai.

[21] Kim SH, Kim YD, Lim DS, Yoon MH, Ahn YK, On YK (2007). Results of a phase III, 8-week, multicenter, prospective, randomized, double-blind, parallel-group clinical trial to assess the effects of amlodipine camsylate versus amlodipine besylate in Korean adults with mild to moderate hypertension. Clin Ther.

[22] Lee HL, Kan CD, Yang YJ (2005). Efficacy and tolerability of the switch from a branded to a generic warfarin sodium product: an observer-blinded, randomized, crossover study. Clin Ther.

[23] Martin BK, Uihlein M, Ings RM, Stevens LA, McEwen J (1984). Comparative bioavailability of two furosemide formulations in humans. J Pharm Sci.

[24] Meyer BH, Muller FO, Swart KJ, Luus HG, Werkman IM (1985). Comparative bio-availability of four formulations of furosemide. S Afr Med J.

[25] Mignini F, Tomassoni D, Traini E, Amenta F (2007). Single-dose, randomized, crossover bioequivalence study of amlodipine maleate versus amlodipine besylate in healthy volunteers. Clin Exp Hypertens.

[26] Mirfazaelian A, Tabatabaeifar M, Rezaee S, Mahmoudian M (2003). Bioequivalence study of atenolol. Daru J Fac Pharm.

[27] Murray MD, Haag KM, Black PK, Hall SD, Brater DC (1997). Variable furosemide absorption and poor predictability of response in elderly patients. Pharmacotherapy.

[28] Neutel JM, Smith DH (1998). A randomized crossover study to compare the efficacy and tolerability of Barr warfarin sodium to the currently available Coumadin. Cardiovasc Rev Rep.

[29] Pan HY, Wang RY, Chan TK (1984). Efficacy of two proprietary preparations of frusemide in patients with congestive heart failure. Med J Aust.

[30] Park JY, Kim KA, Lee GS, Park PW, Kim SL, Lee YS (2004). Randomized, open-label, two-period crossover comparison of the pharmacokinetic and pharmacodynamic properties of two amlodipine formulations in healthy adult male Korean subjects. Clin Ther.

[31] Pereira JA, Holbrook AM, Dolovich L, Goldsmith C, Thabane L, Douketis JD (2005). Are brand-name and generic warfarin interchangeable? Multiple n-of-1 randomized, crossover trials. Ann Pharmacother.

[32] Portoles A, Filipe A, Almeida S, Terleira A, Vallee F, Vargas E (2005). Bioequivalence study of two different tablet formulations of carvedilol in healthy volunteers. Arzneimittelforschung.

[33] Portoles A, Terleira A, Almeida S, Garcia-Arenillas M, Caturla MC, Filipe A (2004). Bioequivalence study of two formulations of enalapril, at a single oral dose of 20 mg (tablets): a randomized, two-way, open-label, crossover study in healthy volunteers. Curr Ther Res Clin Exp.

[34] Rao TR, Usha PR, Naidu MU, Gogtay JA, Meena M (2003). Bioequivalence and tolerability study of two brands of clopidogrel tablets, using inhibition of platelet aggregation and pharmacodynamic measures. Curr Ther Res Clin Exp.

[35] Sarkar MA, Noonan PK, Adams MJ, O’Donnell JP (1995). Pharmacodynamic and pharmacokinetic comparisons to evaluate bioequivalence of atenolol. Clin Res Regul Aff.

[36] Saseen JJ, Porter JA, Barnette DJ, Bauman JL, Zajac EJ, Carter BL (1997). Postabsorption concentration peaks with brand-name and generic verapamil: a double-blind, crossover study in elderly hypertensive patients. J Clin Pharmacol.

[37] Sharoky M, Perkal M, Tabatznik B, Cane RC, Costello K, Goodwin P (1989). Comparative efficacy and bioequivalence of a brand-name and a generic triamterene–hydrochlorothiazide combination product. Clin Pharm.

[38] Singh A, Gupta U, Sagar S, Garg SK, Sharma BK, Mathur VS (1987). Comparative bioequivalence study of furosemide in patients with edema of renal origin. Int J Clin Pharmacol Ther Toxicol.

[39] Tsai YS, Lan SK, Ou JH, Tzai TS (2007). Effects of branded versus generic terazosin hydrochloride in adults with benign prostatic hyperplasia: a randomized, open-label, crossover study in Taiwan. Clin Ther.

[40] Usha PR, Naidu MUR, Kumar TR, Shobha JC, Vijay T (1997). Bioequivalence study of two slow-release diltiazem formulations using dynamic measures in healthy volunteers. Clin Drug Investig.

[41] Waldman SA, Morganroth J (1995). Effects of food on the bioequivalence of different verapamil sustained-release formulations. J Clin Pharmacol.

[42] Weibert RT, Yeager BF, Wittkowsky AK, Bussey HI, Wilson DB, Godwin JE (2000). A randomized, crossover comparison of warfarin products in the treatment of chronic atrial fibrillation. Ann Pharmacother.

[43] Wiwanitkit V, Wangsaturaka D, Tangphao O (2002). LDL-cholesterol lowering effect of a generic product of simvastatin compared to simvastatin (Zocor) in Thai hypercholesterolemic subjects: a randomized crossover study, the first report from Thailand. BMC Clin Pharmacol.

[44] Maitai CK, Ogeto JO, Munenge RW, Ochieng S, Juma FD (1984). A comparative study of the efficacy of seven brands of frusemide tablets. East Afr Med J.

[45] Merali RM, Walker SE, Paton TW, Sheridan BL, Borst SI (1996). Bioavailability and platelet function effect of acetylsalicylic acid. Can J Clin Pharmacol.

[46] Kasmer RJ, Nara AR, Green JA, Chawla AK, Fleming GM (1987). Comparable steady-state bioavailability between two preparations of conventional-release procainamide hydrochloride. Drug Intell Clin Pharm.

[47] Higgins JPT, Green S (2011). Cochrane handbook for systematic reviews of interventions.

[48] Dunlap WP, Cortina JM, Vaslow JB, Burke MJ (1996). Meta-analysis of experiments with matched groups or repeated measures designs. Psychol Methods.

[49] Rosenthal R, Rubin DB (1982). Comparing effect sizes of independent studies. Psychol Bull.

[50] Chinn S (2000). A simple method for converting an odds ratio to effect size for use in meta-analysis. Stat Med.

[51] Kripalani S, Yao X, Haynes RB (2007). Interventions to enhance medication adherence in chronic medical conditions: a systematic review. Arch Intern Med.

[52] Pizzi C, Rutjes AW, Costa GM, Fontana F, Mezzetti A, Manzoli L (2011). Meta-analysis of selective serotonin reuptake inhibitors in patients with depression and coronary heart disease. Am J Cardiol.

[53] Cuadrado A, Rodriguez Gascon A, Hernandez RM, Castilla AM, de la Maza A, Lopez de Ocariz A (2002). In vitro and in vivo equivalence of two oral atenolol tablet formulations. Arzneimittelforschung.

[54] Jia JY, Zhang MQ, Liu YM, Liu Y, Liu GY, Li SJ (2010). Pharmacokinetics and bioequivalence evaluation of two losartan potassium 50-mg tablets: a single-dose, randomized-sequence, open-label, two-way crossover study in healthy Chinese male volunteers. Clin Ther.

[55] Kim SD, Kang W, Lee HW, Park DJ, Ahn JH, Kim MJ (2009). Bioequivalence and tolerability of two clopidogrel salt preparations, besylate and bisulfate: a randomized, open-label, crossover study in healthy Korean male subjects. Clin Ther.

[56] Carranza MJ, Alvarado JMN, Aguirre CIA (2005). Therapeutic equivalence of only dose of three presentations of captopril in women with essential hypertension. Medicina Interna de Mexico.

[57] Higgins JP, Thompson SG, Deeks JJ, Altman DG (2003). Measuring inconsistency in meta-analyses. BMJ.

[58] Ioannidis JP, Trikalinos TA (2007). The appropriateness of asymmetry tests for publication bias in meta-analyses: a large survey. CMAJ Can Med Assoc J..

[59] Friedrich JO, Adhikari NK, Beyene J (2007). Inclusion of zero total event trials in meta-analyses maintains analytic consistency and incorporates all available data. BMC Med Res Methodol.

[60] Lau J, Ioannidis JP, Schmid CH (1997). Quantitative synthesis in systematic reviews. Ann Intern Med.

[61] Carlson JD. NCT01149473 (Losartan potassium/hydrochlorothiazide 100/25 mg tablets in healthy subjects under non-fasting conditions). ClinicalTrialsgov. 2010.

[62] Carlson JD. NCT01149486 (Losartan potassium/hydrochlorothiazide 100/25 mg tablets in healthy subjects under fasting conditions). ClinicalTrialsgov. 2010.

[63] Iqbal M, Khuroo A, Batolar LS, Tandon M, Monif T, Sharma PL (2010). Pharmacokinetics and bioequivalence study of three oral formulations of valsartan 160 mg: a single-dose, randomized, open-label, three-period crossover comparison in healthy Indian male volunteers. Clin Ther.

[64] Kim SH, Chung WY, Zo JH, Kim MA, Chang HJ, Cho YS (2009). Efficacy and tolerability of two formulations of ramipril in Korean adults with mild to moderate essential hypertension: an 8-week, multicenter, prospective, randomized, open-label, parallel-group noninferiority trial. Clin Ther.

[65] Larouche R. NCT01124162 (Losartan 100 mg tablets in healthy subjects under fasting conditions). ClinicalTrialsgov. 2010.

[66] Larouche R. NCT01124175 (Losartan 100 mg tablet in healthy subjects under non-fasting conditions). ClinicalTrialsgov. 2010.

[67] Li KY, Liang JP, Hu BQ, Qiu Y, Luo CH, Jiang Y (2010). The relative bioavailability and fasting pharmacokinetics of three formulations of olmesartan medoxomil 20-mg capsules and tablets in healthy Chinese male volunteers: an open-label, randomized-sequence, single-dose, three-way crossover study. Clin Ther.

[68] Oigman W, Gomes MA, Pereira-Barretto AC, Povoa R, Kohlmann O, Rocha JC (2013). Efficacy and safety of two ramipril and hydrochlorothiazide fixed-dose combination formulations in adults with stage 1 or stage 2 arterial hypertension evaluated by using ABPM. Clin Ther.

[69] Spinola AC, Almeida S, Filipe A, Neves R, Trabelsi F, Farre A (2009). Results of a single-center, single-dose, randomized-sequence, open-label, two-way crossover bioequivalence study of two formulations of valsartan 160-mg tablets in healthy volunteers under fasting conditions. Clin Ther.

[70] Di Girolamo G, Czerniuk P, Bertuola R, Keller GA (2010). Bioequivalence of two tablet formulations of clopidogrel in healthy Argentinian volunteers: a single-dose, randomized-sequence, open-label crossover study. Clin Ther.

[71] Khosravi AR, Pourmoghadas M, Ostovan M, Mehr GK, Gharipour M, Zakeri H (2011). The impact of generic form of Clopidogrel on cardiovascular events in patients with coronary artery stent: results of the OPCES study. J Res Med Sci.

[72] Komosa A, Siller-Matula JM, Kowal J, Lesiak M, Siniawski A, Maczynski M (2014). Comparison of the antiplatelet effect of two clopidogrel bisulfate formulations: plavix and generic-Egitromb. Platelets.

[73] Mijares M, Gomez M, Quijada A, Borges R, Ruiz-Saez A (2008). Eficacia comparativa de dos presentaciones de clopidogrel en la inhibiciòn de la agregaciòn plaquetaria. Arch Venezo Farmacol Terap.

[74] Muller A, Octavio J, Gonzalez MY, Contreras J, Mendez G, Portillo M (2010). Clinical bioequivalence of a dose of clopidogrel Leti Cravid tablets 75 mg versus clopidogrel Sanofi Plavix tablets 75 mg administered on a daily dose for 7 days on healthy volunteers: a clinical trial. Am J Ther.

[75] Oberhansli M, Lehner C, Puricel S, Lehmann S, Togni M, Stauffer JC (2012). A randomized comparison of platelet reactivity in patients after treatment with various commercial clopidogrel preparations: the CLO-CLO trial. Arch Cardiovasc Dis.

[76] Park JB, Koo BK, Choi WG, Kim SY, Park J, Kwan J et al. Comparison of antiplatelet efficacy and tolerability of clopidogrel napadisilate with clopidogrel bisulfate in coronary artery disease patients after percutaneous coronary intervention: a prospective, multicenter, randomized, open-label, phase IV, noninferiority trial. Clin Ther. 2013;35(1):28–37e4. doi:10.1016/j.clinthera.2012.12.004. 10.1016/j.clinthera.2012.12.004 23328268

[77] Seo KW, Tahk SJ, Yang HM, Yoon MH, Shin JH, Choi SY (2014). Point-of-care measurements of platelet inhibition after clopidogrel loading in patients with acute coronary syndrome: comparison of generic and branded clopidogrel bisulfate. Clin Ther.

[78] Srimahachota S, Rojnuckarin P, Udayachalerm W, Buddhari W, Chaipromprasit J, Lertsuwunseri V (2012). Comparison of original and generic clopidogrel 600 mg loading dose in the patients who planned undergoing coronary angiography. J Med Assoc Thai.

[79] Tsoumani ME, Kalantzi KI, Dimitriou AA, Ntalas IV, Goudevenos IA, Tselepis AD (2012). Antiplatelet efficacy of long-term treatment with clopidogrel besylate in patients with a history of acute coronary syndrome: comparison with clopidogrel hydrogen sulfate. Angiology.

[80] Tsoumani ME, Kalantzi KI, Dimitriou AA, Ntalas IV, Goudevenos IA, Tselepis AD (2012). Effect of clopidogrel besylate on platelet reactivity in patients with acute coronary syndromes: comparison with clopidogrel hydrogen sulfate. Expert Opin Pharmacother.

[81] Zou JJ, Tan J, Fan HW, Chen SL (2012). Bioequivalence study of clopidogrel 75 mg tablets in healthy male volunteers. J Bioequiv Bioavailab.

[82] Biswas NR, Garg SK, Kumar N, Mukherjee S, Sharma PL (1989). Comparative pharmacokinetic and pharmacodynamic study of four different brands of propranolol in normal volunteers. Int J Clin Pharmacol Ther Toxicol.

[83] Bus-Kwasnik K, Ksycinska H, Les A, Serafin-Byczak K, Rudzki PJ, Raszek J (2012). Bioequivalence and pharmacokinetics of two 10-mg bisoprolol formulations as film-coated tablets in healthy white volunteers: a randomized, crossover, open-label, 2-period, single-dose, fasting study. Int J Clin Pharmacol Ther.

[84] Liu Y, Lu C, Chen Q, Wang W, Liu GY, Lu XP (2013). Bioequivalence and pharmacokinetic evaluation of two tablet formulations of carvedilol 25-mg: a single-dose, randomized-sequence, open-label, two-way crossover study in healthy Chinese male volunteers. Drug Res (Stuttg).

[85] Kim SA, Park S, Chung N, Lim DS, Yang JY, Oh BH (2008). Efficacy and safety profiles of a new S(−)-amlodipine nicotinate formulation versus racemic amlodipine besylate in adult Korean patients with mild to moderate hypertension: an 8-week, multicenter, randomized, double-blind, double-dummy, parallel-group, phase III, noninferiority clinical trial. Clin Ther.

[86] Liu Y, Jia J, Liu G, Li S, Lu C, Yu C (2009). Pharmacokinetics and bioequivalence evaluation of two formulations of 10-mg amlodipine besylate: an open-label, single-dose, randomized, two-way crossover study in healthy Chinese male volunteers. Clin Ther.

[87] Almeida S, Pedroso P, Filipe A, Pinho C, Neves R, Jimenez C (2011). Study on the bioequivalence of two formulations of eplerenone in healthy volunteers under fasting conditions: data from a single-center, randomized, single-dose, open-label, 2-way crossover bioequivalence study. Arzneimittelforschung.

[88] Kumar S, Monif T, Khuroo A, Reyar S, Jain R, Singla AK (2014). Pharmacokinetic comparison and bioequivalence evaluation of losartan/hydrochlorothiazide tablet between Asian Indian and Japanese volunteers. Int J Clin Pharmacol Ther.

[89] Boh M, Opolski G, Poredos P, Ceska R, Jezovnik M (2011). Therapeutic equivalence of the generic and the reference atorvastatin in patients with increased coronary risk. Int Angiol.

[90] Feng L, Shen-Tu J, Liu J, Chen J, Wu L, Huang M (2009). Bioequivalence of generic and branded subcutaneous enoxaparin: a single-dose, randomized-sequence, open-label, two-period crossover study in healthy Chinese male subjects. Clin Ther.

[91] Kim SH, Park K, Hong SJ, Cho YS, Sung JD, Moon GW (2010). Efficacy and tolerability of a generic and a branded formulation of atorvastatin 20 mg/d in hypercholesterolemic Korean adults at high risk for cardiovascular disease: a multicenter, prospective, randomized, double-blind, double-dummy clinical trial. Clin Ther.

[92] Kim SH, Seo MK, Yoon MH, Choi DH, Hong TJ, Kim HS (2013). Assessment of the efficacy and tolerability of 2 formulations of atorvastatin in Korean adults with hypercholesterolemia: a multicenter, prospective, open-label, randomized trial. Clin Ther.

[93] Liu YM, Pu HH, Liu GY, Jia JY, Weng LP, Xu RJ (2010). Pharmacokinetics and bioequivalence evaluation of two different atorvastatin calcium 10-mg tablets: a single-dose, randomized-sequence, open-label, two-period crossover study in healthy fasted Chinese adult males. Clin Ther.

[94] Palmer JL, Kunhihitlu A, Costantini A, Esquivel F, Roush J, Edwards K, Hill TWK (2014). Pharmacokinetic bioequivalence crossover study of branded generic and innovator formulations of the cholesterol lowering agent ezetimibe. Clin Pharmacol Drug Dev.

[95] Shim CY, Park S, Song JW, Lee SH, Kim JS, Chung N (2010). Comparison of effects of two different formulations of clopidogrel bisulfate tablets on platelet aggregation and bleeding time in healthy Korean volunteers: a single-dose, randomized, open-label, 1-week, two-period, phase IV crossover study. Clin Ther.

[96] Suh JW, Seung KB, Gwak CH, Kim KS, Hong SJ, Park TH (2011). Comparison of antiplatelet effect and tolerability of clopidogrel resinate with clopidogrel bisulfate in patients with coronary heart disease (CHD) or CHD-equivalent risks: a phase IV, prospective, double-dummy, parallel-group, 4-week noninferiority trial. Clin Ther.

[97] Shrank WH, Cox ER, Fischer MA, Mehta J, Choudhry NK (2009). Patients’ perceptions of generic medications. Health Aff (Millwood).

[98] Shrank WH, Liberman JN, Fischer MA, Girdish C, Brennan TA, Choudhry NK (2011). Physician perceptions about generic drugs. Ann Pharmacother.

[99] Himmel W, Simmenroth-Nayda A, Niebling W, Ledig T, Jansen RD, Kochen MM (2005). What do primary care patients think about generic drugs?. Int J Clin Pharmacol Ther.

[100] Corrao G, Soranna D, Arfe A, Casula M, Tragni E, Merlino L (2014). Are generic and brand-name statins clinically equivalent? Evidence from a real data-base. Eur J Intern Med..

[101] Gagne JJ, Choudhry NK, Kesselheim AS, Polinski JM, Hutchins D, Matlin OS (2014). Comparative effectiveness of generic and brand-name statins on patient outcomes: a cohort study. Ann Intern Med.

[102] IMS Health. Generic Medicines: Essential contributors to the long-term health of society. London: IMS Health; 2013.

[103] OECD (2013). Pharmaceutical generic market share, in Health at a Glance 2013: OECD indicators.

[104] The Global Use of Medicines: outlook through 2017. IMS Institute for Healthcare Informatics. 2013. http://www.imshealth.com/cds/imshealth/Global/Content/Corporate/IMS%20Health%20Institute/Reports/Global_Use_of_Meds_Outlook_2017/IIHI_Global_Use_of_Meds_Report_2013.pdf. Accessed December 2014.

[105] The Cardiovascular Market Outlook to 2016. Business insights. 2011. http://download.bioon.com.cn/view/upload/201303/16084307_4163.pdf. Accessed December 2014.

[106] Nilsen V, Bakke PS, Gallefoss F (2011). Effects of lifestyle intervention in persons at risk for type 2 diabetes mellitus: results from a randomised, controlled trial. BMC Public Health.

[107] Manzoli L, Flacco ME, D’Addario M, Capasso L, De Vito C, Marzuillo C (2014). Non-publication and delayed publication of randomized trials on vaccines: survey. BMJ.

[108] International Committee of Medical Journal Editors (ICMJE). Recommendations for the conduct, reporting, editing, and publication of scholarly work in medical journals. 2014. http://www.icmje.org/icmje-recommendations.pdf. 25558501

[109] Faasse K, Cundy T, Gamble G, Petrie KJ (2013). The effect of an apparent change to a branded or generic medication on drug effectiveness and side effects. Psychosom Med.

[110] Alpern JD, Stauffer WM, Kesselheim AS (2014). High-cost generic drugs-implications for patients and policymakers. N Engl J Med.

[111] Shrank WH, Choudhry NK, Liberman JN, Brennan TA (2011). The use of generic drugs in prevention of chronic disease is far more cost-effective than thought, and may save money. Health Aff (Millwood).

[112] Jackevicius CA, Chou MM, Ross JS, Shah ND, Krumholz HM (2012). Generic atorvastatin and health care costs. N Engl J Med.

