# Supplementary material for: Generic versus brand-name drugs used in cardiovascular diseases
Source: Eur J Epidemiol. 2015 Nov 30;31:351–68. doi: 10.1007/s10654-015-0104-8 (PMC4877434; doi:10.1007/s10654-015-0104-8)
Supplement: Supplementary file 1 — Supplementary material 1 (DOC 1190 kb) [file 10654_2015_104_MOESM1_ESM.doc]

**Additional files captions**

**Additional Appendix S1**. Search string details.

**Additional Appendix S2**. List of the excluded trials, and reasons for the exclusion.

**Additional Table S1**. List of the clinical outcomes reported by each included trial.

**Additional Table S2**. Methodological characteristics of included studies: (+) indicates low risk of bias, (?) unclear and (-) a high risk of bias on a specific item .

**Additional Table S3**. Results of the meta-regression predicting the summary estimate of efficacy (none of the variables that have not been included in the final model were significant).

**Additional Table S4**. Results of the meta-regression predicting the summary estimate of mild/moderate adverse events (none of the variables that have not been included in the final model were significant).

**Additional Appendix S1. Search string details**

**Search strings used in PubMed - Investigator 1**

**Box 1:** Boolean search terms and algorithms used to search PubMed

| **generic-name drugs** |
| --- |
| #1 Drugs, Generic/therapeutic use[Mesh Terms/Subheading]  #2 Drugs, Generic/adverse effects[Mesh Terms/Subheading]  #3 generic*[Title/Abstract]  #4 nonproprietary[Title/Abstract]  #5 non-brand[Title/Abstract] |
| **brand-name drugs** |
| #6 brand [Title/Abstract]  #7 proprietary[Title/Abstract]  #8 innovator[Title/Abstract]  #9 trade[Title/Abstract] |
| **drugs** |
| #10 drug*[Title/Abstract]  #11 preparation*[Title/Abstract]  #12 formulation*[Title/Abstract]  #13 medication*[Title/Abstract] |
| **cardiovascular drugs** |
| #14 Cardiovascular agents[Mesh Terms]  #15 antihypertensive*[All fields]  #16 antiarrhythmic*[All fields]  #17 beta blocker*[All fields]  #18 calcium channel blocker*[All fields]  #19 ace inhibitor*[All fields]  #20 diuretic*[All fields]  #21 statin*[All fields] |

| **Algorithms** |
| --- |
| (#1 OR #2) AND #6; (#1 OR #2) AND #7; (#1 OR #2) AND #8; (#1 OR #2) AND #9; #3 AND #6; #4 AND #6; #5 AND #6; #3 AND #7; #4 AND #7; #5 AND #7; #3 AND #8; #4 AND #8; #5 AND #8; #3 AND #9; #3 AND #10; #3 AND #11; #3 AND #12; #3 AND #13; #6 AND #10; #6 AND #11; #6 AND #12; #6 AND #13; #7 AND #10; #7 AND #11; #7 AND #12; #7 AND #13; #14 AND #3 AND #6; #14 AND #3 AND #7; #14 AND #3 AND #8; #15 AND #3; #15 AND #4; #15 AND #5; #15 AND #6; #15 AND #7; #15 AND #8; #15 AND #9; #16 AND #3; #16 AND #4; #16 AND #5; #16 AND #6; #16 AND #7; #16 AND #8; #16 AND #9; #17 AND #3; #17 AND #4; #17 AND #5; #17 AND #6; #17 AND #7; #17 AND #8; #17 AND #9; #18 AND #3; #18 AND #4; #18 AND #5; #18 AND #6; #18 AND #7; #18 AND #8; #18 AND #9; #19 AND #3; #19 AND #4; #19 AND #5; #19 AND #6; #19 AND #7; #19 AND #8; #19 AND #9; #20 AND #3; #20 AND #4; #20 AND #5; #20 AND #6; #20 AND #7; #20 AND #8; #20 AND #9; #21 AND #3; #21 AND #4; #21 AND #5; #21 AND #6; #21 AND #7; #21 AND #8; #21 AND #9  *Filters: Clinical Trials; Review; Systematic Reviews* |

**Search strings used in PubMed - Investigator 2**

(drugs, generic[MeSH Terms]) AND brand[Title/Abstract]

(drugs, generic[MeSH Terms]) AND proprietary[Title/Abstract]

(drugs, generic[MeSH Terms]) AND innovator[Title/Abstract]

(drugs, generic[MeSH Terms]) AND formulation[Title/Abstract]

(drugs, generic[MeSH Terms]) AND trade[Title/Abstract]

(generic[Title/Abstract]) AND brand[Title/Abstract]

(generic[Title/Abstract]) AND preparation[Title/Abstract]

(generic[Title/Abstract]) AND formulation[Title/Abstract]

(generic[Title/Abstract]) AND medication[Title/Abstract]

(generic[Title/Abstract]) AND proprietary[Title/Abstract]

(generic[Title/Abstract]) AND innovator[Title/Abstract]

(generic[Title/Abstract]) AND trade[Title/Abstract]

(non-proprietary[Title/Abstract]) AND brand[Title/Abstract]

(non-proprietary[Title/Abstract]) AND proprietary[Title/Abstract]

(non-proprietary[Title/Abstract]) AND innovator[Title/Abstract]

(non-proprietary[Title/Abstract]) AND trade[Title/Abstract]

(non-proprietary[Title/Abstract]) AND drug[Title/Abstract]

(non-proprietary[Title/Abstract]) AND formulation[Title/Abstract]

(non-proprietary[Title/Abstract]) AND preparation[Title/Abstract]

(non-proprietary[Title/Abstract]) AND medication[Title/Abstract]

(non-brand[Title/Abstract]) AND brand[Title/Abstract]

(non-brand[Title/Abstract]) AND proprietary[Title/Abstract]

(non-brand[Title/Abstract]) AND innovator[Title/Abstract]

(non-brand[Title/Abstract]) AND trade[Title/Abstract]

(non-brand[Title/Abstract]) AND drug[Title/Abstract]

(non-brand[Title/Abstract]) AND preparation[Title/Abstract]

(non-brand[Title/Abstract]) AND medication[Title/Abstract]

(non-brand[Title/Abstract]) AND formulation[Title/Abstract]

((agents, cardiovascular[MeSH Terms]) AND generic[Title/Abstract]) AND brand[Title/Abstract]

((agents, cardiovascular[MeSH Terms]) AND generic[Title/Abstract]) AND preparation[Title/Abstract]

((agents, cardiovascular[MeSH Terms]) AND generic[Title/Abstract]) AND formulation[Title/Abstract]

((agents, cardiovascular[MeSH Terms]) AND generic[Title/Abstract]) AND medication[Title/Abstract]

(antihypertensive) AND generic[Title/Abstract]

(antihypertensive) AND proprietary[Title/Abstract]

(antihypertensive) AND brand[Title/Abstract]

(antihypertensive) AND innovator[Title/Abstract]

(antihypertensive) AND trade[Title/Abstract]

(antiarrhythmic) AND proprietary[Title/Abstract]

(antiarrhythmic) AND generic[Title/Abstract]

(beta blocker) AND generic[Title/Abstract]

(beta blocker) AND proprietary[Title/Abstract]

(beta blocker) AND non-brand[Title/Abstract]

(beta blocker) AND innovator[Title/Abstract]

(beta blocker) AND formulation[Title/Abstract]

(calcium channel blocker) AND generic[Title/Abstract]

(calcium channel blocker) AND brand[Title/Abstract]

(calcium channel blocker) AND formulation[Title/Abstract]

(ace inhibitor) AND generic[Title/Abstract]

(ace inhibitor) AND formulation[Title/Abstract]

(ace inhibitor) AND preparation[Title/Abstract]

(diuretic) AND generic[Title/Abstract]

(diuretic) AND preparation[Title/Abstract]

(diuretic) AND formulation[Title/Abstract]

(statin) AND generic[Title/Abstract]

(statin) AND preparation[Title/Abstract]

**Search strings used in Scopus - Investigator 2**

ALL ( drug ) AND TITLE-ABS-KEY ( randomized ) AND TITLE-ABS-KEY ( generic ) AND TITLE-ABS-KEY ( brand ) AND ( LIMIT-TO ( PUBYEAR , 2015 ) OR LIMIT-TO ( PUBYEAR , 2014 ) OR LIMIT-TO ( PUBYEAR , 2013 ) OR LIMIT-TO ( PUBYEAR , 2012 ) OR LIMIT-TO ( PUBYEAR , 2011 ) OR LIMIT-TO ( PUBYEAR , 2010 ) OR LIMIT-TO ( PUBYEAR , 2009 ) OR LIMIT-TO ( PUBYEAR , 2008 ) OR LIMIT-TO ( PUBYEAR , 2007 ) OR LIMIT-TO ( PUBYEAR , 2006 ) ) AND ( LIMIT-TO ( DOCTYPE , "ar" ) ) AND ( EXCLUDE ( SUBJAREA , "HEAL" ) OR EXCLUDE ( SUBJAREA , "SOCI" ) ) AND ( EXCLUDE ( SUBJAREA , "BUSI" ) ) AND ( EXCLUDE ( SUBJAREA , "ECON" ) OR EXCLUDE ( SUBJAREA , "PHYS" ) OR EXCLUDE ( SUBJAREA , "AGRI" ) ) AND ( EXCLUDE ( SUBJAREA , "ARTS" ) )

ALL ( drug ) AND TITLE-ABS-KEY ( randomized ) AND TITLE-ABS-KEY ( proprietary ) AND ( LIMIT-TO ( PUBYEAR , 2014 ) OR LIMIT-TO ( PUBYEAR , 2013 ) OR LIMIT-TO ( PUBYEAR , 2012 ) OR LIMIT-TO ( PUBYEAR , 2011 ) OR LIMIT-TO ( PUBYEAR , 2010 ) OR LIMIT-TO ( PUBYEAR , 2009 ) OR LIMIT-TO ( PUBYEAR , 2008 ) OR LIMIT-TO ( PUBYEAR , 2007 ) OR LIMIT-TO ( PUBYEAR , 2006 ) ) AND ( LIMIT-TO ( DOCTYPE , "ar" ) OR LIMIT-TO ( DOCTYPE , "ip" ) ) )

ALL ( drug ) AND TITLE-ABS-KEY ( randomized ) AND TITLE-ABS-KEY ( brand ) AND ( LIMIT-TO ( PUBYEAR , 2014 ) OR LIMIT-TO ( PUBYEAR , 2013 ) OR LIMIT-TO ( PUBYEAR , 2012 ) OR LIMIT-TO ( PUBYEAR , 2011 ) OR LIMIT-TO ( PUBYEAR , 2010 ) OR LIMIT-TO ( PUBYEAR , 2009 ) OR LIMIT-TO ( PUBYEAR , 2008 ) OR LIMIT-TO ( PUBYEAR , 2007 ) OR LIMIT-TO ( PUBYEAR , 2006 ) ) AND ( LIMIT-TO ( DOCTYPE , "ar" ) OR LIMIT-TO ( DOCTYPE , "ip" ) ) )

ALL ( drug ) AND TITLE-ABS-KEY ( randomized ) AND TITLE-ABS-KEY ( innovator ) AND ( LIMIT-TO ( PUBYEAR , 2014 ) OR LIMIT-TO ( PUBYEAR , 2013 ) OR LIMIT-TO ( PUBYEAR , 2012 ) OR LIMIT-TO ( PUBYEAR , 2011 ) OR LIMIT-TO ( PUBYEAR , 2010 ) OR LIMIT-TO ( PUBYEAR , 2009 ) OR LIMIT-TO ( PUBYEAR , 2008 ) OR LIMIT-TO ( PUBYEAR , 2007 ) OR LIMIT-TO ( PUBYEAR , 2006 ) ) AND ( LIMIT-TO ( DOCTYPE , "ar" ) OR LIMIT-TO ( DOCTYPE , "ip" ) ) )

ALL ( drug ) AND TITLE-ABS-KEY ( randomized ) AND TITLE-ABS-KEY ( trade ) AND ( LIMIT-TO ( PUBYEAR , 2014 ) OR LIMIT-TO ( PUBYEAR , 2013 ) OR LIMIT-TO ( PUBYEAR , 2012 ) OR LIMIT-TO ( PUBYEAR , 2011 ) OR LIMIT-TO ( PUBYEAR , 2010 ) OR LIMIT-TO ( PUBYEAR , 2009 ) OR LIMIT-TO ( PUBYEAR , 2008 ) OR LIMIT-TO ( PUBYEAR , 2007 ) OR LIMIT-TO ( PUBYEAR , 2006 ) ) AND ( LIMIT-TO ( DOCTYPE , "ar" ) OR LIMIT-TO ( DOCTYPE , "ip" ) ) )

ALL ( drug ) AND ALL ( antihypertensive ) AND TITLE-ABS-KEY ( randomized ) AND ALL ( generic ) AND ( LIMIT-TO ( PUBYEAR , 2014 ) OR LIMIT-TO ( PUBYEAR , 2013 ) OR LIMIT-TO ( PUBYEAR , 2012 ) OR LIMIT-TO ( PUBYEAR , 2011 ) OR LIMIT-TO ( PUBYEAR , 2010 ) OR LIMIT-TO ( PUBYEAR , 2009 ) OR LIMIT-TO ( PUBYEAR , 2008 ) OR LIMIT-TO ( PUBYEAR , 2007 ) OR LIMIT-TO ( PUBYEAR , 2006 ) ) AND ( LIMIT-TO ( DOCTYPE , "ar" ) ) )

ALL ( drug ) AND ALL ( antihypertensive ) AND TITLE-ABS-KEY ( randomized ) AND ALL ( brand ) AND ( LIMIT-TO ( PUBYEAR , 2014 ) OR LIMIT-TO ( PUBYEAR , 2013 ) OR LIMIT-TO ( PUBYEAR , 2012 ) OR LIMIT-TO ( PUBYEAR , 2011 ) OR LIMIT-TO ( PUBYEAR , 2010 ) OR LIMIT-TO ( PUBYEAR , 2009 ) OR LIMIT-TO ( PUBYEAR , 2008 ) OR LIMIT-TO ( PUBYEAR , 2007 ) OR LIMIT-TO ( PUBYEAR , 2006 ) ) AND ( LIMIT-TO ( DOCTYPE , "ar" ) ) )

ALL ( drug ) AND ALL ( antihypertensive ) AND TITLE-ABS-KEY ( randomized ) AND ALL ( proprietary ) AND ( LIMIT-TO ( PUBYEAR , 2014 ) OR LIMIT-TO ( PUBYEAR , 2013 ) OR LIMIT-TO ( PUBYEAR , 2012 ) OR LIMIT-TO ( PUBYEAR , 2011 ) OR LIMIT-TO ( PUBYEAR , 2010 ) OR LIMIT-TO ( PUBYEAR , 2009 ) OR LIMIT-TO ( PUBYEAR , 2008 ) OR LIMIT-TO ( PUBYEAR , 2007 ) OR LIMIT-TO ( PUBYEAR , 2006 ) ) AND ( LIMIT-TO ( DOCTYPE , "ar" ) ) )

ALL ( drug ) AND ALL ( antihypertensive ) AND TITLE-ABS-KEY ( randomized ) AND ALL ( trade ) AND ( LIMIT-TO ( PUBYEAR , 2014 ) OR LIMIT-TO ( PUBYEAR , 2013 ) OR LIMIT-TO ( PUBYEAR , 2012 ) OR LIMIT-TO ( PUBYEAR , 2011 ) OR LIMIT-TO ( PUBYEAR , 2010 ) OR LIMIT-TO ( PUBYEAR , 2009 ) OR LIMIT-TO ( PUBYEAR , 2008 ) OR LIMIT-TO ( PUBYEAR , 2007 ) OR LIMIT-TO ( PUBYEAR , 2006 ) ) AND ( LIMIT-TO ( DOCTYPE , "ar" ) ) )

ALL ( drug ) AND ALL ( antiarrhythmic ) AND TITLE-ABS-KEY ( randomized ) AND ALL ( generic ) AND ( LIMIT-TO ( PUBYEAR , 2014 ) OR LIMIT-TO ( PUBYEAR , 2013 ) OR LIMIT-TO ( PUBYEAR , 2012 ) OR LIMIT-TO ( PUBYEAR , 2011 ) OR LIMIT-TO ( PUBYEAR , 2010 ) OR LIMIT-TO ( PUBYEAR , 2009 ) OR LIMIT-TO ( PUBYEAR , 2008 ) OR LIMIT-TO ( PUBYEAR , 2007 ) OR LIMIT-TO ( PUBYEAR , 2006 ) ) AND ( LIMIT-TO ( DOCTYPE , "ar" ) ) )

ALL ( drug ) AND ALL ( antiarrhythmic ) AND TITLE-ABS-KEY ( randomized ) AND ALL ( brand ) AND ( LIMIT-TO ( PUBYEAR , 2014 ) OR LIMIT-TO ( PUBYEAR , 2013 ) OR LIMIT-TO ( PUBYEAR , 2012 ) OR LIMIT-TO ( PUBYEAR , 2011 ) OR LIMIT-TO ( PUBYEAR , 2010 ) OR LIMIT-TO ( PUBYEAR , 2009 ) OR LIMIT-TO ( PUBYEAR , 2008 ) OR LIMIT-TO ( PUBYEAR , 2007 ) OR LIMIT-TO ( PUBYEAR , 2006 ) ) AND ( LIMIT-TO ( DOCTYPE , "ar" ) ) )

ALL ( drug ) AND ALL ( antiarrhythmic ) AND TITLE-ABS-KEY ( randomized ) AND ALL ( proprietary ) AND ( LIMIT-TO ( PUBYEAR , 2014 ) OR LIMIT-TO ( PUBYEAR , 2013 ) OR LIMIT-TO ( PUBYEAR , 2012 ) OR LIMIT-TO ( PUBYEAR , 2011 ) OR LIMIT-TO ( PUBYEAR , 2010 ) OR LIMIT-TO ( PUBYEAR , 2009 ) OR LIMIT-TO ( PUBYEAR , 2008 ) OR LIMIT-TO ( PUBYEAR , 2007 ) OR LIMIT-TO ( PUBYEAR , 2006 ) ) AND ( LIMIT-TO ( DOCTYPE , "ar" ) ) )

ALL ( drug ) AND ALL ( antiarrhythmic ) AND TITLE-ABS-KEY ( randomized ) AND ALL ( trade ) AND ( LIMIT-TO ( PUBYEAR , 2014 ) OR LIMIT-TO ( PUBYEAR , 2013 ) OR LIMIT-TO ( PUBYEAR , 2012 ) OR LIMIT-TO ( PUBYEAR , 2011 ) OR LIMIT-TO ( PUBYEAR , 2010 ) OR LIMIT-TO ( PUBYEAR , 2009 ) OR LIMIT-TO ( PUBYEAR , 2008 ) OR LIMIT-TO ( PUBYEAR , 2007 ) OR LIMIT-TO ( PUBYEAR , 2006 ) ) AND ( LIMIT-TO ( DOCTYPE , "ar" ) ) )

ALL ( drug ) AND ALL ( beta blocker ) AND TITLE-ABS-KEY ( randomized ) AND ALL ( generic ) AND ( LIMIT-TO ( PUBYEAR , 2014 ) OR LIMIT-TO ( PUBYEAR , 2013 ) OR LIMIT-TO ( PUBYEAR , 2012 ) OR LIMIT-TO ( PUBYEAR , 2011 ) OR LIMIT-TO ( PUBYEAR , 2010 ) OR LIMIT-TO ( PUBYEAR , 2009 ) OR LIMIT-TO ( PUBYEAR , 2008 ) OR LIMIT-TO ( PUBYEAR , 2007 ) OR LIMIT-TO ( PUBYEAR , 2006 ) ) AND ( LIMIT-TO ( DOCTYPE , "ar" ) ) )

ALL ( drug ) AND ALL ( beta blocker ) AND TITLE-ABS-KEY ( randomized ) AND ALL ( brand ) AND ( LIMIT-TO ( PUBYEAR , 2014 ) OR LIMIT-TO ( PUBYEAR , 2013 ) OR LIMIT-TO ( PUBYEAR , 2012 ) OR LIMIT-TO ( PUBYEAR , 2011 ) OR LIMIT-TO ( PUBYEAR , 2010 ) OR LIMIT-TO ( PUBYEAR , 2009 ) OR LIMIT-TO ( PUBYEAR , 2008 ) OR LIMIT-TO ( PUBYEAR , 2007 ) OR LIMIT-TO ( PUBYEAR , 2006 ) ) AND ( LIMIT-TO ( DOCTYPE , "ar" ) ) )

ALL ( drug ) AND ALL ( beta blocker ) AND TITLE-ABS-KEY ( randomized ) AND ALL ( proprietary ) AND ( LIMIT-TO ( PUBYEAR , 2014 ) OR LIMIT-TO ( PUBYEAR , 2013 ) OR LIMIT-TO ( PUBYEAR , 2012 ) OR LIMIT-TO ( PUBYEAR , 2011 ) OR LIMIT-TO ( PUBYEAR , 2010 ) OR LIMIT-TO ( PUBYEAR , 2009 ) OR LIMIT-TO ( PUBYEAR , 2008 ) OR LIMIT-TO ( PUBYEAR , 2007 ) OR LIMIT-TO ( PUBYEAR , 2006 ) ) AND ( LIMIT-TO ( DOCTYPE , "ar" ) ) )

ALL ( drug ) AND ALL ( beta blocker ) AND TITLE-ABS-KEY ( randomized ) AND ALL ( trade ) AND ( LIMIT-TO ( PUBYEAR , 2014 ) OR LIMIT-TO ( PUBYEAR , 2013 ) OR LIMIT-TO ( PUBYEAR , 2012 ) OR LIMIT-TO ( PUBYEAR , 2011 ) OR LIMIT-TO ( PUBYEAR , 2010 ) OR LIMIT-TO ( PUBYEAR , 2009 ) OR LIMIT-TO ( PUBYEAR , 2008 ) OR LIMIT-TO ( PUBYEAR , 2007 ) OR LIMIT-TO ( PUBYEAR , 2006 ) ) AND ( LIMIT-TO ( DOCTYPE , "ar" ) ) )

ALL ( drug ) AND ALL ( calcium channel blocker ) AND TITLE-ABS-KEY ( randomized ) AND ALL ( generic ) AND ( LIMIT-TO ( PUBYEAR , 2014 ) OR LIMIT-TO ( PUBYEAR , 2013 ) OR LIMIT-TO ( PUBYEAR , 2012 ) OR LIMIT-TO ( PUBYEAR , 2011 ) OR LIMIT-TO ( PUBYEAR , 2010 ) OR LIMIT-TO ( PUBYEAR , 2009 ) OR LIMIT-TO ( PUBYEAR , 2008 ) OR LIMIT-TO ( PUBYEAR , 2007 ) OR LIMIT-TO ( PUBYEAR , 2006 ) ) AND ( LIMIT-TO ( DOCTYPE , "ar" ) ) )

ALL ( drug ) AND ALL ( calcium channel blocker ) AND TITLE-ABS-KEY ( randomized ) AND ALL ( brand ) AND ( LIMIT-TO ( PUBYEAR , 2014 ) OR LIMIT-TO ( PUBYEAR , 2013 ) OR LIMIT-TO ( PUBYEAR , 2012 ) OR LIMIT-TO ( PUBYEAR , 2011 ) OR LIMIT-TO ( PUBYEAR , 2010 ) OR LIMIT-TO ( PUBYEAR , 2009 ) OR LIMIT-TO ( PUBYEAR , 2008 ) OR LIMIT-TO ( PUBYEAR , 2007 ) OR LIMIT-TO ( PUBYEAR , 2006 ) ) AND ( LIMIT-TO ( DOCTYPE , "ar" ) ) )

ALL ( drug ) AND ALL ( calcium channel blocker ) AND TITLE-ABS-KEY ( randomized ) AND ALL ( proprietary ) AND ( LIMIT-TO ( PUBYEAR , 2014 ) OR LIMIT-TO ( PUBYEAR , 2013 ) OR LIMIT-TO ( PUBYEAR , 2012 ) OR LIMIT-TO ( PUBYEAR , 2011 ) OR LIMIT-TO ( PUBYEAR , 2010 ) OR LIMIT-TO ( PUBYEAR , 2009 ) OR LIMIT-TO ( PUBYEAR , 2008 ) OR LIMIT-TO ( PUBYEAR , 2007 ) OR LIMIT-TO ( PUBYEAR , 2006 ) ) AND ( LIMIT-TO ( DOCTYPE , "ar" ) ) )

ALL ( drug ) AND ALL ( calcium channel blocker ) AND TITLE-ABS-KEY ( randomized ) AND ALL ( trade ) AND ( LIMIT-TO ( PUBYEAR , 2014 ) OR LIMIT-TO ( PUBYEAR , 2013 ) OR LIMIT-TO ( PUBYEAR , 2012 ) OR LIMIT-TO ( PUBYEAR , 2011 ) OR LIMIT-TO ( PUBYEAR , 2010 ) OR LIMIT-TO ( PUBYEAR , 2009 ) OR LIMIT-TO ( PUBYEAR , 2008 ) OR LIMIT-TO ( PUBYEAR , 2007 ) OR LIMIT-TO ( PUBYEAR , 2006 ) ) AND ( LIMIT-TO ( DOCTYPE , "ar" ) ) )

ALL ( drug ) AND ALL ( ace inhibitor ) AND TITLE-ABS-KEY ( randomized ) AND ALL ( generic ) AND ( LIMIT-TO ( PUBYEAR , 2014 ) OR LIMIT-TO ( PUBYEAR , 2013 ) OR LIMIT-TO ( PUBYEAR , 2012 ) OR LIMIT-TO ( PUBYEAR , 2011 ) OR LIMIT-TO ( PUBYEAR , 2010 ) OR LIMIT-TO ( PUBYEAR , 2009 ) OR LIMIT-TO ( PUBYEAR , 2008 ) OR LIMIT-TO ( PUBYEAR , 2007 ) OR LIMIT-TO ( PUBYEAR , 2006 ) ) AND ( LIMIT-TO ( DOCTYPE , "ar" ) ) )

ALL ( drug ) AND ALL ( ace inhibitor ) AND TITLE-ABS-KEY ( randomized ) AND ALL ( brand ) AND ( LIMIT-TO ( PUBYEAR , 2014 ) OR LIMIT-TO ( PUBYEAR , 2013 ) OR LIMIT-TO ( PUBYEAR , 2012 ) OR LIMIT-TO ( PUBYEAR , 2011 ) OR LIMIT-TO ( PUBYEAR , 2010 ) OR LIMIT-TO ( PUBYEAR , 2009 ) OR LIMIT-TO ( PUBYEAR , 2008 ) OR LIMIT-TO ( PUBYEAR , 2007 ) OR LIMIT-TO ( PUBYEAR , 2006 ) ) AND ( LIMIT-TO ( DOCTYPE , "ar" ) ) )

ALL ( drug ) AND ALL ( diuretic ) AND TITLE-ABS-KEY ( randomized ) AND ALL ( generic ) AND ( LIMIT-TO ( PUBYEAR , 2014 ) OR LIMIT-TO ( PUBYEAR , 2013 ) OR LIMIT-TO ( PUBYEAR , 2012 ) OR LIMIT-TO ( PUBYEAR , 2011 ) OR LIMIT-TO ( PUBYEAR , 2010 ) OR LIMIT-TO ( PUBYEAR , 2009 ) OR LIMIT-TO ( PUBYEAR , 2008 ) OR LIMIT-TO ( PUBYEAR , 2007 ) OR LIMIT-TO ( PUBYEAR , 2006 ) ) AND ( LIMIT-TO ( DOCTYPE , "ar" ) ) )

ALL ( drug ) AND ALL ( diuretic ) AND TITLE-ABS-KEY ( randomized ) AND ALL ( brand ) AND ( LIMIT-TO ( PUBYEAR , 2014 ) OR LIMIT-TO ( PUBYEAR , 2013 ) OR LIMIT-TO ( PUBYEAR , 2012 ) OR LIMIT-TO ( PUBYEAR , 2011 ) OR LIMIT-TO ( PUBYEAR , 2010 ) OR LIMIT-TO ( PUBYEAR , 2009 ) OR LIMIT-TO ( PUBYEAR , 2008 ) OR LIMIT-TO ( PUBYEAR , 2007 ) OR LIMIT-TO ( PUBYEAR , 2006 ) ) AND ( LIMIT-TO ( DOCTYPE , "ar" ) ) )

ALL ( drug ) AND ALL ( diuretic ) AND TITLE-ABS-KEY ( randomized ) AND ALL ( trade ) AND ( LIMIT-TO ( PUBYEAR , 2014 ) OR LIMIT-TO ( PUBYEAR , 2013 ) OR LIMIT-TO ( PUBYEAR , 2012 ) OR LIMIT-TO ( PUBYEAR , 2011 ) OR LIMIT-TO ( PUBYEAR , 2010 ) OR LIMIT-TO ( PUBYEAR , 2009 ) OR LIMIT-TO ( PUBYEAR , 2008 ) OR LIMIT-TO ( PUBYEAR , 2007 ) OR LIMIT-TO ( PUBYEAR , 2006 ) ) AND ( LIMIT-TO ( DOCTYPE , "ar" ) ) )

ALL ( drug ) AND ALL ( statin ) AND TITLE-ABS-KEY ( randomized ) AND ALL ( generic ) AND ( LIMIT-TO ( PUBYEAR , 2014 ) OR LIMIT-TO ( PUBYEAR , 2013 ) OR LIMIT-TO ( PUBYEAR , 2012 ) OR LIMIT-TO ( PUBYEAR , 2011 ) OR LIMIT-TO ( PUBYEAR , 2010 ) OR LIMIT-TO ( PUBYEAR , 2009 ) OR LIMIT-TO ( PUBYEAR , 2008 ) OR LIMIT-TO ( PUBYEAR , 2007 ) OR LIMIT-TO ( PUBYEAR , 2006 ) ) AND ( LIMIT-TO ( DOCTYPE , "ar" ) ) )

ALL ( drug ) AND ALL ( statin ) AND TITLE-ABS-KEY ( randomized ) AND ALL ( brand ) AND ( LIMIT-TO ( PUBYEAR , 2014 ) OR LIMIT-TO ( PUBYEAR , 2013 ) OR LIMIT-TO ( PUBYEAR , 2012 ) OR LIMIT-TO ( PUBYEAR , 2011 ) OR LIMIT-TO ( PUBYEAR , 2010 ) OR LIMIT-TO ( PUBYEAR , 2009 ) OR LIMIT-TO ( PUBYEAR , 2008 ) OR LIMIT-TO ( PUBYEAR , 2007 ) OR LIMIT-TO ( PUBYEAR , 2006 ) ) AND ( LIMIT-TO ( DOCTYPE , "ar" ) ) )

ALL ( drug ) AND ALL ( statin ) AND TITLE-ABS-KEY ( randomized ) AND ALL ( trade ) AND ( LIMIT-TO ( PUBYEAR , 2014 ) OR LIMIT-TO ( PUBYEAR , 2013 ) OR LIMIT-TO ( PUBYEAR , 2012 ) OR LIMIT-TO ( PUBYEAR , 2011 ) OR LIMIT-TO ( PUBYEAR , 2010 ) OR LIMIT-TO ( PUBYEAR , 2009 ) OR LIMIT-TO ( PUBYEAR , 2008 ) OR LIMIT-TO ( PUBYEAR , 2007 ) OR LIMIT-TO ( PUBYEAR , 2006 ) ) AND ( LIMIT-TO ( DOCTYPE , "ar" ) ) )

ALL ( drug ) AND ALL ( statin ) AND TITLE-ABS-KEY ( randomized ) AND ALL ( proprietary ) AND ( LIMIT-TO ( PUBYEAR , 2014 ) OR LIMIT-TO ( PUBYEAR , 2013 ) OR LIMIT-TO ( PUBYEAR , 2012 ) OR LIMIT-TO ( PUBYEAR , 2011 ) OR LIMIT-TO ( PUBYEAR , 2010 ) OR LIMIT-TO ( PUBYEAR , 2009 ) OR LIMIT-TO ( PUBYEAR , 2008 ) OR LIMIT-TO ( PUBYEAR , 2007 ) OR LIMIT-TO ( PUBYEAR , 2006 ) ) AND ( LIMIT-TO ( DOCTYPE , "ar" ) ) )

**Additional Appendix S2**. List of the excluded trials, and reasons for the exclusion.

| **First author** | **Year** | **Drug class** | **Reason for the exclusion** |
| --- | --- | --- | --- |
|  |  |  |  |
| *Bioequivalence data only* | | | |
| Niopas | 2003 | ACE-Inhibitors | Bioequivalence outcomes only |
| Bienert | 2006 | ACE-Inhibitors | Bioequivalence outcomes only |
| Perez | 2006 | ACE-Inhibitors | Bioequivalence outcomes only |
| Shin | 2008 | ACE-Inhibitors | Bioequivalence outcomes only |
| Cohen Sabban | 2009 | ACE-Inhibitors | Bioequivalence outcomes only |
| Zakeri-Milani | 2010 | ACE-Inhibitors | Bioequivalence outcomes only |
| Wittayalertpanya | 2014 | ACE-Inhibitors | Bioequivalence outcomes. Some relevant outcomes were mentioned, but no data could be extracted |
| Zaid | 2014 | ACE-Inhibitors | Bioequivalence outcomes only |
| Yacobi | 2000 | Anticoagulants | Bioequivalence outcomes only |
| Merali | 1996 | Antiplatelet | Bioequivalence outcomes. Some relevant outcomes were mentioned, but no data could be extracted |
| Lainesse | 2004 | Antiplatelet | Bioequivalence outcomes only |
| El Ahmady | 2009 | Antiplatelet | Bioequivalence outcomes only |
| Filipe | 2009 | Antiplatelet | Bioequivalence outcomes only |
| Richter | 2009 | Antiplatelet | Bioequivalence outcomes only |
| Khan | 2010 | Antiplatelet | Bioequivalence outcomes. Some relevant outcomes were mentioned, but no data could be extracted |
| Setiawati | 2011 | Antiplatelet | Bioequivalence outcomes only |
| Sambu | 2012 | Antiplatelet | Bioequivalence outcomes only |
| Eldon | 1989 | Beta-blockers | Bioequivalence outcomes only |
| Rostok | 1995 | Beta-blockers | Bioequivalence outcomes only |
| Yuen | 1998 | Beta-blockers | Bioequivalence outcomes only |
| Peh | 1999 | Beta-blockers | Bioequivalence outcomes only |
| Rojanasthien | 1999 | Beta-blockers | Bioequivalence outcomes only |
| Jovanovic | 2006 | Beta-blockers | Bioequivalence outcomes only |
| Carter | 1993 | Calcium Channel Blockers | Bioequivalence outcomes. Some relevant outcomes were mentioned, but no data could be extracted |
| Waldman | 1995 | Calcium Channel Blockers | Bioequivalence outcomes. Some relevant outcomes were mentioned, but no data could be extracted |
| Tsang | 1996 | Calcium Channel Blockers | Bioequivalence outcomes only |
| Rojanasthien | 2004 | Calcium Channel Blockers | Bioequivalence outcomes only |
| Sailer | 2007 | Calcium Channel Blockers | Bioequivalence outcomes only |
| Barbhaiya | 1982 | Diuretics | Bioequivalence outcomes only |
| Maitai | 1984 | Diuretics | Bioequivalence outcomes. Some relevant outcomes were mentioned, but no data could be extracted |
| Shah | 1984 | Diuretics | Bioequivalence outcomes only |
| Hamidi | 2011 | Diuretics | Bioequivalence outcomes only |
| Shaw | 1973 | Others | Bioequivalence outcomes only |
| Zinny | 1984 | Others | Bioequivalence outcomes only |
| Hilleman | 1988 | Others | Bioequivalence outcomes only |
| Valecha | 1993 | Others | Bioequivalence outcomes only |
| Huang | 2013 | Others | Bioequivalence outcomes only |
| Najib | 2003 | Statins | Bioequivalence outcomes only |
|  |  |  |  |
| *Other reasons* | | | |
| Mulla | 2010 | ACE-Inhibitors | Different formulations of the same drug |
| Gasche | 2013 | Antiplatelet | Two different drugs |
| Theidel | 2013 | Antiplatelet | Two different drugs |
| Faasse | 2013 | Beta-blockers | Only a placebo drug was used |
| Brown | 2008 | Calcium Channel Blockers | Different formulations of the same drug |
| Roca-Cusachs | 2008 | Diuretics | Different formulations of the same drug |
| Kasmer | 1987 | Others | Two different drugs |
| Ghaffari | 2011 | Others | Different formulations of the same drug |
| Arsenault | 2012 | Others | One drug is no more sold. Besides, data could not be extracted. |
| McPherson | 2001 | Statins | Two different drugs |
| Park | 2005 | Statins | Two different drugs |
| Rodriguez Roa | 2007 | Statins | Different formulations of the same drug |
| Tran | 2007 | Statins | Two different drugs |
| Ballantyne | 2008 | Statins | Two different drugs |
| Insull | 2009 | Statins | Two different drugs |
| Rodriguez Roa | 2010 | Statins | Different formulations of the same drug |
|  |  |  |  |

**Additional Table S1**. List of the clinical outcomes reported by each included trial. Each outcome was classified as: SoE = Soft efficacy outcome; HaE = Hard efficacy outcome; H1 = mild/moderate adverse event/harm; H2 = serious adverse event/harm. Each comparison was classified as: (+) favoring generic drug; (-) favoring brand-name drug; (=) non significant differences detected; NR = no results available.

| **Study** | **Outcome(s)** | **Type** | **Data extraction** | **Significance** | **Notes** |
| --- | --- | --- | --- | --- | --- |
|  |  |  |  |  |  |
| *ACE inhibitors or ARBs* |  |  |  |  |  |
|  |  |  |  |  |  |
| Portoles 2004 | - SBP  - DBP  - Heart rate  - Cell blood count  - Urinalysis  - mAEs (dizziness, fatigue, headache,  somnolence)  - sAEs (not further specified) | - SoE  - SoE  - SoE  - SoE  - SoE  - H1  - H2 | - Extracted  - Extractable  - Not extractable  - Not extractable  - Not extractable  - Extracted  - Extracted | =  =  NR  =  =  =  = | Efficacy data were recorded at 5, 12, 24 and 36 hours. Based upon the estimated duration of the effect, we extracted data at the 5-hour time-point. |
| Carranza 2005 | - SBP  - DBP  - Heart rate | - SoE  - SoE  - SoE | - Extracted  - Extractable  - Extractable | =  =  = | The data for two doses (25 mg and 50 mg) of both generic and brand-name captopril were reported. We extracted 50 mg data. |
| Kim (A) 2009 | - SBP  - DBP  - Pulse wave velocity  - Exercise capacity  - Left ventricular diastolic function  - Brain natriuretic peptide  - C-reactive protein  - mAEs (cough, dizziness, headache,  fatigue, abdominal pain, pruritus)  - sAEs (not further specified) | - SoE  - SoE  - SoE  - SoE  - SoE  - SoE  - SoE  - H1  - H2 | - Extracted  - Extractable  - Extractable  - Extractable  - Extractable  - Extractable  - Extractable  - Extracted  - Extracted | =  =  =  +  =  =  =  =  = |  |
| Spinola 2009 | - ECG  - Urinalysis  - mAEs (BP decrease, dizziness, headache, fatigue, pruritus)  - sAEs (not further specified) | - SoE  - SoE  - H1  - H2 | - Not extractable  - Not extractable  - Extracted  - Extracted | NR  NR  =  = |  |
| Iqbal 2010 | - Mean blood pressure  - sAEs (not further specified) | - SoE  - H2 | - Not extractable  - Extracted | NR  = |  |
| Jia 2010 | - SBP  - DBP  - Heart rate  - mAEs (hematuria)  - sAEs (not further specified) | - SoE  - SoE  - SoE  - H1  - H2 | - Extracted  - Extractable  - Extractable  - Extracted  - Extracted | =  =  =  =  = | Efficacy data were recorded at 1, 2, 3, 4, 5, 6, 12, 24 and 36 hours. Based upon the estimated duration of the effect, we extracted data at the 24-hour time-point. |
| Li 2010 | - Mean blood pressure  - Heart rate  - Respiratory rate  - sAEs (not further specified) | - SoE  - SoE  - SoE  - H2 | - Not extractable  - Not extractable  - Not extractable  - Extracted | NR  NR  NR  = |  |
| Larouche (1) 2010 | - mAEs (low blood pressure, headache, dizziness)  - sAEs | - H1  - H2 | - Extracted  - Extracted | -  = | Results posted in ClinicalTrials.gov only |
| Larouche (2) 2010 | - mAEs (low blood pressure)  - sAEs | - H1  - H2 | - Extracted  - Extracted | -  = | Results posted in ClinicalTrials.gov only |
| Carlson (1) 2010 | - sAEs | - H2 | - Extracted | = | Results posted in ClinicalTrials.gov only |
| Carlson (2) 2010 | - mAEs (headache, dizziness, sweat, pharyngitis, purpura, confusion)  - sAEs | - H1  - H2 | - Extracted  - Extracted | +  = | Results posted in ClinicalTrials.gov only |
| Oigman 2013 | - SBP  - DBP  - 24-hour ambulatory blood pressure  - Cell blood count  - Urinalysis  - mAEs (cough, headache, dizziness, palpitation)  - sAEs | - SoE  - SoE  - SoE  - SoE  - SoE  - H1  - H2 | - Extracted  - Extractable  - Extractable  - Not extractable  - Not extractable  - Extracted  - Extracted | =  =  =  NR  NR  =  = |  |
|  |  |  |  |  |  |
| *Anticoagulants* |  |  |  |  |  |
|  |  |  |  |  |  |
| Handler 1998 | - % within INR range  - mAEs (ecchymosis, epistaxis, gum hemorrhage, rectal hemorrhage)  - sAEs (not further specified) | - SoE  - H1  - H2 | - Extracted  - Extracted  - Extracted | =  =  = |  |
| Neutel 1998 | - % within INR range  - mAEs | - SoE  - H1 | - Extracted  - Not extractable | =  NR |  |
| Weibert 2000 | - % within INR range  - Drug dosage change  - mAEs (anticoagulation events)  - sAEs (not further specified) | - SoE  - SoE  - H1  - H2 | - Not extractable  - Not extractable  - Extracted  - Extracted | =  =  =  = |  |
| Lee 2005 | - % within INR range  - Protrombin time  - mAEs (ecchymosis, epistaxis, gum hemorrhage, rectal hemorrhage)  - sAEs | - SoE  - SoE  - H1  - H2 | - Extracted  - Not extractable  - Extracted  - Extracted | =  NR  =  = |  |
| Pereira 2005 | - % within INR range  - Protrombin time | - SoE  - SoE | - Extracted  - Not extractable | =  NR |  |
|  |  |  |  |  |  |
| *Antiplatelet agents* |  |  |  |  |  |
|  |  |  |  |  |  |
| Rao 2003 | - Bleeding time  - Platelet aggregation inhibition  - Platelet count  - Neutrophil count  - mAEs (headache)  - sAEs (not further specified) | - SoE  - SoE  - SoE  - SoE  - H1  - H2 | - Extracted  - Extractable  - Not extractable  - Not extractable  - Extracted  - Extracted | =  =  NR  NR  =  = | The total length of follow-up was 10 days, but the extracted efficacy outcome was measured after 2 hours. |
| Ashraf 2005 | - Platelet aggregation inhibition  - ECG  - Creatine kinase  - Liver function tests  - mAEs (headache, dizziness, weakness)  - sAEs (not further specified) | - SoE  - SoE  - SoE  - SoE  - H1  - H2 | - Extracted  - Not extractable  - Not extractable  - Not extractable  - Not extractable  - Extracted | =  NR  NR  NR  NR  = |  |
| Mijares 2008 ***** | - Platelet aggregation inhibition  - mAEs (headache, hematoma, ecchymosis, pruritus)  - sAEs (not further specified) | - SoE  - H1  - H2 | - Extracted  - Extracted  - Extracted | =  -  = |  |
| Kim (P) 2009 | - Platelet aggregation inhibition  - Protrombin time  - Bleeding time  - Cell blood count  - mAEs (abdominal pain)  - sAEs (not further specified) | - SoE  - SoE  - SoE  - SoE  - H1  - H2 | - Extracted  - Not extractable  - Not extractable  - Not extractable  - Extracted  - Extracted | =  NR  NR  NR  =  = | The total length of follow-up was 13 days, but the extracted efficacy outcome was measured after 7 hours. |
| Di Girolamo 2010 | - sAEs (not further specified) | - H2 | - Extracted | = |  |
| Muller 2010 | - Platelet aggregation inhibition | - SoE | - Extracted | = |  |
| Shim 2010 | - Bleeding time  - Platelet aggregation inhibition  - Mean blood pressure  - Heart rate  - Cell blood count  - Liver function tests  - sAEs (not further specified) | - SoE  - SoE  - SoE  - SoE  - SoE  - SoE  - H2 | - Extracted  - Extractable  - Not extractable  - Not extractable  - Not extractable  - Not extractable  - Extracted | =  =  =  =  =  NR  = |  |
| Khosravi 2011 | - MACE and death  - Angina episodes  - Bleeding  - Platelet count  - Neutrophil count  - Liver function tests | - HaE  - HaE  - SoE  - SoE  - SoE  - SoE | - Extracted  - NR  - Not extractable  - Not extractable  - Not extractable  - Not extractable | =  NR  =  =  =  = |  |
| Suh 2011 | - mAEs (not further specified)  - sAEs (stable angina) | - H1  - H2 | - Extracted  - Extracted | =  = |  |
| Oberhänsli 2012 * | - Platelet aggregation inhibition  - Platelet count  - Protrombin time  - Activated partial thromboplastin time  - sAEs (not further specified) | - SoE  - SoE  - SoE  - SoE  - H2 | - Extracted  - Not extractable  - Not extractable  - Not extractable  - Extracted | =  =  =  =  = |  |
| Srimahachota 2012 ***** | - Platelet aggregation inhibition  - mAEs (hematoma)  - sAEs (not further specified) | - SoE  - H1  - H2 | - Extracted  - Extracted  - Extracted | =  =  = |  |
| Tsoumani (A) 2012 | - Platelet aggregation inhibition | - SoE | - Extracted | = |  |
| Tsoumani (E) 2012 | - Platelet aggregation inhibition | - SoE | - Extracted | = |  |
| Zou 2012 ***** | - sAEs (not further specified) | - H2 | - Extracted | = |  |
| Park 2013 | - MACE and death  - Platelet aggregation inhibition  - mAEs (hematoma, pruritus, rash, urticaria)  - sAEs (not further specified) | - HaE  - SoE  - H1  - H2 | - Extracted  - Extracted  - Extracted  - Extracted | =  =  =  = |  |
| Komosa 2014 ***** | - Platelet aggregation inhibition  - mAEs (hematoma) | -.SoE  - H1 | - Extracted  - Extracted | =  = |  |
| Seo 2014 | - MACE and death  - Platelet aggregation inhibition  - sAEs | - HaE  - SoE  - H2 | - Extracted  - Extracted  - Extracted | =  =  = | Data on platelet aggregation were recorded at 2, 4, 8 and 24 hours. Based upon the estimated duration of the effect, we extracted data at the 24-hour time-point. Data on MACE and death were reported at 4-weeks time-point only. |
|  |  |  |  |  |  |
| *Beta-blockers* |  |  |  |  |  |
|  |  |  |  |  |  |
| Biswas 1989 | - SBP  - DBP  - Heart rate | - SoE  - SoE  - SoE | - Extracted  - Extractable  - Extractable | =  =  = |  |
| Carter 1989 | - SBP  - DBP  - Heart rate | - SoE  - SoE  - SoE | - Extracted  - Extractable  - Extractable | =  =  = |  |
| el-Sayed 1989 | - SBP  - Heart rate | - SoE  - SoE | - Extracted  - Extractable | =  = |  |
| Chiang 1995 | - SBP  - DBP | - SoE  - SoE | - Extracted  - Extractable | =  = |  |
| Sarkar 1995 | - SBP  - DBP  - Heart rate  - sAEs (not further specified) | - SoE  - SoE  - SoE  - H2 | - Extracted  - Extractable  - Extractable  - Extracted | =  =  =  = | Efficacy data were recorded up to 24 hours. Based upon the estimated duration of the effect, we extracted data at the 12-hour time-point. |
| Bongers 1999 | - SBP  - DBP  - Heart rate  - Heart rate variability  - ST-segment depression magnitude  - Extrasystoles frequency  - Number of angina attacks  - mAEs (headache)  - sAEs (not further specified) | - SoE  - SoE  - SoE  - SoE  - SoE  - SoE  - SoE  - H1  - H2 | - Extracted  - Extractable  - Extractable  - Not extractable  - Extractable  - Not extractable  - Extractable  - Extracted  - Extracted | =  =  +  =  =  =  =  =  = |  |
| Cuadrado 2002 | - SBP  - DBP  - Heart rate  - mAEs (dizziness, pirosis, headache)  - sAEs (not further specified) | - SoE  - SoE  - SoE  - H1  - H2 | - Extracted  - Extractable  - Extractable  - Extracted  - Extracted | =  =  =  =  = | Efficacy data were recorded up to 30 hours. Based upon the estimated duration of the effect, we extracted data at the 24-hour time-point. |
| Mirfazaelian 2003 | - SBP  - DBP  - Heart rate  - sAEs (not further specified) | - SoE  - SoE  - SoE  - H2 | - Extracted  - Extractable  - Extractable  - Extracted | =  =  =  = |  |
| Portoles 2005 | - SBP  - DBP  - Heart rate  - mAEs (headache, dizziness, diarrhea)  - sAEs (not further specified) | - SoE  - SoE  - SoE  - H1  - H2 | - Not extractable  - Not extractable  - Not extractable  - Not extractable  - Extracted | =  =  =  =  = |  |
| Bus-Kwasnik 2012 * | - Mean blood pressure  - Heart rate  - mAEs (headache, vomiting)  - sAEs (not further specified) | - SoE  - SoE  - H1  - H2 | - Not extractable  - Not extractable  - Extracted  - Extracted | NR  NR  =  = |  |
| Liu 2013 | - mAEs (headache, dizziness, diarrhea)  - sAEs (not further specified) | - H1  - H2 | - Extracted  - Extracted | =  = |  |
|  |  |  |  |  |  |
| *Calcium channel blockers* |  |  |  |  |  |
|  |  |  |  |  |  |
| Saseen 1997 | - SBP  - DBP  - Heart rate  - PR interval prolongation  - mAEs  - sAEs (atrio-ventricular block) | - SoE  - SoE  - SoE  - SoE  - H1  - H2 | - Extracted  - Extractable  - Extractable  - Extractable  - Not extractable  - Extracted | =  =  =  =  NR  = |  |
| Usha 1997 | - SBP  - DBP  - Heart rate  - PQ interval prolongation  - sAEs (not further specified) | - SoE  - SoE  - SoE  - SoE  - H2 | - Extracted  - Extractable  - Extractable  - Not extractable  - Not extractable | =  =  =  =  = | Efficacy data were recorded up to 24 hours. Based upon the estimated duration of the effect, we extracted data at the 12-hour time-point. |
| Park 2004 | - SBP  - DBP  - Heart rate  - Hematology testing  - Biochemistry testing  - Urinalysis  - sAEs (not further specified) | - SoE  - SoE  - SoE  - SoE  - SoE  - SoE  - H2 | - Extracted  - Extractable  - Extractable  - Not extractable  - Not extractable  - Not extractable  - Extracted | =  =  =  =  =  =  = | The total length of follow-up was 6 days, but the extracted efficacy outcome was measured after 8 hours. |
| Kim 2007 | - SBP  - DBP  - Heart rate  - Cell blood count  - Biochemistry testing  - mAEs (headache, dizziness, dispnea)  - sAEs (not further specified) | - SoE  - SoE  - SoE  - SoE  - SoE  - H1  - H2 | - Extracted  - Extractable  - Not extractable  - Not extractable  - Not extractable  - Extracted  - Extracted | =  =  NR  =  =  =  = |  |
| Mignini 2007 | - SBP  - DBP  - Heart rate  - ECG  - Hematology testing  - Biochemistry testing  - Liver function test  - mAEs (dizziness, fatigue)  - sAEs (not further specified) | - SoE  - SoE  - SoE  - SoE  - SoE  - SoE  - SoE  - H1  - H2 | - Extracted  - Not extractable  - Extractable  - Not extractable  - Not extractable  - Not extractable  - Not extractable  - Extracted  - Extracted | =  =  =  NR  =  =  =  =  = | The total length of follow-up was 6 days, but the extracted efficacy outcome was measured after 3 hours. |
| Kim 2008 | - SBP  - DBP  - Heart rate  - Serum uric acid levels  - mAEs (headache, dizziness, flushing, chest pain, pedal edema, paresthesia)  - sAEs (not further specified) | - SoE  - SoE  - SoE  - SoE  - H1  - H2 | - Extracted  - Extractable  - Extractable  - Not extractable  - Extracted  - Extracted | =  =  =  =  =  = |  |
| Liu 2009 | - Mean blood pressure  - Heart rate  - Hematology testing  - Biochemistry testing  - Urinalysis  - mAEs (not further specified)  - sAEs (not further specified) | - SoE  - SoE  - SoE  - SoE  - SoE  - H1  - H2 | - Not extractable  - Not extractable  - Not extractable  - Not extractable  - Not extractable  - Extracted  - Extracted | NR  NR  NR  NR  NR  =  = |  |
|  |  |  |  |  |  |
| *Diuretics* |  |  |  |  |  |
|  |  |  |  |  |  |
| Garg 1984 | - SBP  - DBP  - Heart rate  - Electrolyte excretion  - Urine volume  - mAEs (weakness)  - sAEs (not further specified) | - SoE  - SoE  - SoE  - SoE  - SoE  - H1  - H2 | - Extracted  - Extractable  - Extractable  - Extractable  - Extractable  - Extracted  - Extracted | =  =  =  =  =  +  = |  |
| Grahnen 1984 | - Urine volume | - SoE | - Extracted | = |  |
| Martin 1984 | - Sodium excretion  - Urine volume | - SoE  - SoE | - Extracted  - Extractable | =  = |  |
| Pan 1984 | - Sodium excretion  - Urine volume  - Serum electrolytes levels  - Serum glucose levels  - Serum uric acid levels  - Mean body weight decrease  - Mean blood pressure  - Heart rate | - SoE  - SoE  - SoE  - SoE  - SoE  - SoE  - SoE  - SoE | - Extracted  - Not extractable  - Extractable  - Not extractable  - Not extractable  - Not extractable  - Not extractable  - Not extractable | =  =  =  =  =  =  =  = |  |
| Meyer 1985 | - Urine volume | - SoE | - Extracted | - |  |
| Singh 1987 | - SBP  - DBP  - Heart rate  - Sodium excretion  - Urine volume  - sAEs (not further specified) | - SoE  - SoE  - SoE  - SoE  - SoE  - H2 | - Extracted  - Extractable  - Extractable  - Extractable  - Extractable  - Extracted | =  =  =  =  =  = |  |
| Sharoky 1989 | - SBP  - DBP  - Serum electrolytes levels  - sAEs (not further specified) | - SoE  - SoE  - SoE  - H2 | - Extracted  - Extractable  - Extractable  - Extracted | =  =  =  = |  |
| Kaojarern 1990 | - Sodium excretion  - Urine volume | - SoE  - SoE | - Extracted  - Not extractable | =  = |  |
| Awad 1992 | - Sodium excretion (=) (E)  - Urine volume (=) | - SoE  - SoE | - Extracted  - Extractable | =  = |  |
| Murray 1997 | - Sodium excretion | - SoE | - Extracted | = |  |
| Almeida 2011 | - Mean blood pressure  - Heart rate  - Hematology testing  - Biochemistry testing  - Urinalysis  - mAEs (hypoesthesia, somnolence)  - sAEs (not further specified) | - SoE  - SoE  - SoE  - SoE  - SoE  - H1  - H2 | - Not extractable  - Not extractable  - Not extractable  - Not extractable  - Not extractable  - Extracted  - Extracted | NR  NR  NR  NR  NR  -  = |  |
| Kumar 2014 (India) | - mAEs (headache, furuncle, vomiting)  - sAEs (not further specified) | - H1  - H2 | - Not extractable  - Extracted | =  = |  |
| Kumar 2014 (Japan) | - sAEs (not further specified) | - H2 | - Extracted | = |  |
|  |  |  |  |  |  |
| *Statins* |  |  |  |  |  |
|  |  |  |  |  |  |
| Assawawitoontip 2002 | - LDL  - Liver function tests  - Creatine kinase | - SoE  - SoE  - SoE | - Extracted  - Not extractable  - Not extractable | =  =  = |  |
| Wiwanitkit 2002 | - LDL  - HDL  - Total cholesterol  - Triglycerides  - Liver function tests  - Creatine kinase  - sAEs (not further specified) | - SoE  - SoE  - SoE  - SoE  - SoE  - SoE  - H2 | - Extracted  - Extractable  - Extractable  - Extractable  - Extractable  - Extractable  - Extracted | =  =  =  =  =  =  = |  |
| Kim 2010 | - LDL  - HDL  - Total cholesterol  - Triglycerides  - A1-B Apolipoproteins  - C reactive protein  -% of patients with LDL<100 mg/dL  - mAEs (cold, gastrointestinal symptoms, arthritis, myalgia)  - sAEs (not further specified) | - SoE  - SoE  - SoE  - SoE  - SoE  - SoE  - SoE  - H1  - H2 | - Extracted  - Extractable  - Extractable  - Extractable  - Extractable  - Extractable  - Extractable  - Extracted  - Extracted | =  =  =  =  =  =  =  -  = |  |
| Liu 2010 | - Mean blood pressure  - Heart rate  - ECG  - Cell blood count  - Urinalysis  - mAEs (abdominal pain, syncope)  - sAEs (not further specified) | - SoE  - SoE  - SoE  - SoE  - SoE  - H1  - H2 | - Not extractable  - Not extractable  - Not extractable  - Not extractable  - Not extractable  - Not extractable  - Extracted | NR  NR  NR  NR  NR  =  = |  |
| Boh 2011 | - LDL  - HDL  - Total cholesterol  - Triglycerides  - A1-B Apolipoproteins  - Liver function tests  - Creatine kinase  - SBP  - DBP  - mAEs (myalgia, lack of appetite)  - sAEs (not further specified | - SoE  - SoE  - SoE  - SoE  - SoE  - SoE  - SoE  - SoE  - SoE  - H1  - H2 | - Extracted  - Extractable  - Extractable  - Extractable  - Extractable  - Not extractable  - Not extractable  - Extractable  - Extractable  - Extracted  - Extracted | =  =  =  =  =  =  =  =  =  =  = |  |
| Kim 2013 | - LDL  - HDL  - Total cholesterol  - Triglycerides  - A1-B Apolipoproteins  - C reactive proteins  - % of patients with LDL <100 mg/dL  - mAEs (cough, myalgia, headache)  - sAEs (toxic hepatitis, chest pain) | - SoE  - SoE  - SoE  - SoE  - SoE  - SoE  - SoE  - H1  - H2 | - Extracted  - Extractable  - Extractable  - Extractable  - Extractable  - Extractable  - Extractable  - Extracted  - Extracted | =  =  =  =  =  =  =  =  = |  |
|  |  |  |  |  |  |
| *Others* |  |  |  |  |  |
|  |  |  |  |  |  |
| Tsai 2007 | - SBP  - DBP  - Heart rate  - Hematology testing  - Biochemistry testing  - Urinalysis  - mAEs (dizziness, peripheral edema)  - sAEs (hospitalization for epididymithis) | - SoE  - SoE  - SoE  - SoE  - SoE  - SoE  - H1  - H2 | - Extracted  - Extractable  - Extractable  - Extractable  - Extractable  - Extractable  - Extracted  - Extracted | =  =  =  =  =  =  =  = |  |
| Feng 2009 | - sAEs (not further specified) | - H2 | - Extracted | = |  |
| Palmer 2014 | - Clinical examination  - Hematology testing  - Biochemistry testing  - sAEs | - SoE  - SoE  - SoE  - H2 | - Not extractable  - Not extractable  - Not extractable  - Extracted | NR  NR  NR  = |  |

SBP = Systolic blood pressure. DBP = Diastolic blood pressure. INR = International normalized ratio. MACE = Major cardiovascular events. LDL = Low Density Lipoprotein Cholesterol. mAEs = Mild or moderate adverse events. sAEs = Serious adverse events.

***** We were able to extract these datasets thanks to the indications of the investigators of other studies.

**Additional Table S2**. Methodological characteristics of included studies: (+) indicates low risk of bias, (?) unclear and (-) a high risk of bias on a specific item .

|  | Adequate  sequence  generation? | Allocation  concealment? | Adequate  blinding of  participants, personnel and outcome assessors? | Incomplete outcome data adequately addressed? | Selective outcome reporting? | Other potential threats to validity? |
| --- | --- | --- | --- | --- | --- | --- |
|  |  |  |  |  |  |  |
| *ACE inhibitors or ARBs* |  |  |  |  |  |  |
| Portoles 2004 | + | - | - | + | + | + |
| Carranza 2005 | + | - | + | + | - | - |
| Kim (A) 2009 | ? | - | - | + | - | - |
| Spinola 2009 | + | + | - | + | + | + |
| Iqbal 2010 | ? | - | - | - | - | - |
| Jia 2010 | + | - | - | + | - | - |
| Li 2010 | + | - | - | + | + | + |
| Oigman 2013 | ? | - | - | + | - | - |
| Larouche (1) 2010 | *NP* | *NP* | *NP* | *NP* | *NP* | *NP* |
| Larouche (2) 2010 | *NP* | *NP* | *NP* | *NP* | *NP* | *NP* |
| Carlson (1) 2010 | *NP* | *NP* | *NP* | *NP* | *NP* | *NP* |
| Carlson (2) 2010 | *NP* | *NP* | *NP* | *NP* | *NP* | *NP* |
|  |  |  |  |  |  |  |
| *Anticoagulants* |  |  |  |  |  |  |
| Handler 1998 | + | - | - | + | + | - |
| Neutel 1998 | ? | ? | - | + | - | + |
| Weibert 2000 | + | - | - | - | - | - |
| Lee 2005 | + | + | - | + | - | - |
| Pereira 2005 | ? | + | + | + | - | - |
|  |  |  |  |  |  |  |
| *Antiplatelet agents* |  |  |  |  |  |  |
| Rao 2003 | + | - | - | + | + | + |
| Ashraf 2005 | ? | - | + | - | - | - |
| Mijares 2008 | ? | ? | ? | + | - | + |
| Kim (P) 2009 | + | - | - | + | - | - |
| Di Girolamo 2010 | + | - | - | + | - | - |
| Müller 2010 | ? | ? | ? | + | + | + |
| Shim 2010 | + | - | - | + | - | - |
| Khosravi 2011 | + | - | + | + | + | + |
| Suh 2011 | + | - | + | + | + | + |
| Oberhänsli 2012 | ? | - | - | + | + | + |
| Srimahachota 2012 | ? | ? | - | + | - | + |
| Tsoumani (A) 2012 | ? | - | - | + | - | - |
| Tsoumani (E) 2012 | - | - | - | + | - | - |
| Zou 2012 | + | ? | - | + | - | + |
| Park (J) 2013 | + | - | - | + | + | - |
| Komosa 2014 | + | ? | ? | + | - | + |
| Seo 2014 | ? | - | - | + | + | - |
|  |  |  |  |  |  |  |
| *Beta-blockers* |  |  |  |  |  |  |
| Biswas 1989 | - | - | - | + | - | - |
| Carter 1989 | + | - | - | + | + | - |
| el-Sayed 1989 | + | - | + | + | - | - |
| Chiang 1995 | ? | ? | + | + | - | + |
| Sarkar 1995 | ? | - | + | + | - | - |
| Bongers 1999 | ? | - | + | + | + | + |
| Cuadrado 2002 | ? | - | - | + | - | - |
| Mirfazaelian 2003 | - | - | + | + | - | - |
| Portoles 2005 | ? | ? | - | + | - | - |
| Bus-Kwasnik 2012 | ? | ? | - | + | + | + |
| Liu 2013 | + | ? | - | + | - | + |
|  |  |  |  |  |  |  |
| *Calcium channel blockers* |  |  |  |  |  |  |
| Saseen 1997 | ? | ? | + | + | - | + |
| Usha 1997 | - | - | + | + | - | - |
| Park 2004 | + | ? | - | + | + | + |
| Kim 2007 | + | + | + | + | + | + |
| Mignini 2007 | + | ? | - | + | - | + |
| Kim 2008 | + | + | + | + | + | + |
| Liu 2009 | + | ? | - | + | - | - |
|  |  |  |  |  |  |  |
| *Diuretics* |  |  |  |  |  |  |
| Garg 1984 | ? | ? | + | + | - | + |
| Grahnen 1984 | ? | - | + | + | + | + |
| Martin 1984 | - | - | - | - | + | - |
| Pan 1984 | - | - | + | ? | + | - |
| Meyer 1985 | ? | - | + | ? | - | - |
| Singh 1987 | ? | - | + | + | - | - |
| Sharoky 1989 | ? | - | + | - | - | - |
| Kaojarern 1990 | ? | ? | - | + | - | + |
| Awad 1992 | - | - | ? | + | - | - |
| Murray 1997 | ? | - | - | + | + | + |
| Almeida 2011 | + | + | ? | + | - | + |
| Kumar (I) 2014 | + | ? | - | + | - | + |
| Kumar (J) 2014 | + | ? | - | + | - | + |
|  |  |  |  |  |  |  |
| *Statins* |  |  |  |  |  |  |
| Assawawitoontip 2002 | ? | + | + | + | - | + |
| Wiwanitkit 2002 | ? | + | + | + | + | + |
| Liu 2010 | + | - | - | + | + | - |
| Boh 2011 | + | + | + | + | + | - |
| Kim 2010 | + | + | + | + | + | + |
| Kim 2013 | + | ? | - | + | + | + |
|  |  |  |  |  |  |  |
| *Others* |  |  |  |  |  |  |
| Tsai 2007 | + | ? | - | + | + | + |
| Feng 2009 | + | - | - | + | - | - |
| Palmer 2014 | + | - | - | + | - | + |
|  |  |  |  |  |  |  |

NP = Not possible; the results have been posted in ClinicalTrials.gov only.

**Additional Table S3**. Results of the meta-regression predicting the summary estimate of efficacy (none of the variables that have not been included in the final model were significant).

|  | Regression  coefficient | 95% CI | p* |
| --- | --- | --- | --- |
|  |  |  |  |
| Unhealthy vs healthy | 0.139 | (-0.029; 0.308) | 0.10 |
| Follow-up duration, 1-day increase | -0.004 | (-0.186; 0.179) | 0.9 |
| Double blinding | -0.005 | (-0.075; 0.064) | 0.9 |
| Sample size, 1-unit increase | -0.001 | (-0.003; 0.001) | 0.2 |
| Funded by generic manufacturers | 0.110 | (-0.046; 0.267) | 0.2 |
|  |  |  |  |

**Additional Table S4**. Results of the meta-regression predicting the summary estimate of mild/moderate adverse events (none of the variables that have not been included in the final model were significant).

|  | Regression  coefficient | 95% CI | p* |
| --- | --- | --- | --- |
|  |  |  |  |
| Unhealthy vs healthy | 0.149 | (-0.537; 0.836) | 0.7 |
| Follow-up duration, 1-day increase | -0.283 | (-0.994; 0.429) | 0.4 |
| Double blinding | 0.178 | (-0.092; 0.449) | 0.2 |
| Sample size, 1-unit increase | -0.003 | (-0.007; 0.001) | 0.2 |
|  |  |  |  |

**References**

1. Higgins JPT, Green, S.,. Cochrane Handbook for Systematic Reviews of Interventions: The Cochrane Collaboration; 2011. Available from: www.cochrane-handbook.org.

2. Niopas I, Daftsios AC, Nikolaidis N. Bioequivalence study of two brands of enalapril tablets after single oral administration to healthy volunteers. Int J Clin Pharmacol Ther. 2003;41(5):226-30.

3. Bienert A, Brzeziniski R, Szalek E, Dubai V, Grzeskowiak E, Dyderski S, et al. Bioequivalence study of two losartan formulations administered orally in healthy male volunteers. Arzneimittelforschung. 2006;56(11):723-8.

4. Pérez M, Cárdenas, W, Ramírez, G, Pérez, M, Restrepo, P. A comparative, cross-over, double blind, randomized study for bioequivalence assessment between two formulations of valsartan capsules vs. tablets. 2006;37(2).

5. Shin MC, Kim JK, Kim CK. Bioequivalence evaluation of two brands of lisinopril tablets by in vitro comparative dissolution test and in vivo bioequivalence test. Arzneimittelforschung. 2008;58(1):11-7.

6. Cohen Sabban H, Gonzalez, Y.R. Evaluacion comparativa de niveles plasmaticos de dos formulaciones de enalapril: Enalapril (Renitec (C)) de Laboratorios Merck Sharp & Dohme vs Enalapril LETI. Archivos Venezolanos de Farmacología y Terapéutica. 2009;28((Suppl. 3)):1-2.

7. Zakeri-Milani P, Valizadeh H, Islambulchilar Z, Nemati M. Pharmacokinetic and bioequivalence study of two brands of valsartan tablets in healthy male volunteers. Arzneimittelforschung. 2010;60(2):76-80.

8. Wittayalertpanya S, Chariyavilaskul P, Prompila N, Sayankuldilok N, Eiamart W. Pharmacokinetics and bioequivalence study of irbesartan tablets after a single oral dose of 300 mg in healthy Thai volunteers. Int J Clin Pharmacol Ther. 2014;52(5):431-6.

9. Zaid AN, Natur S, Qaddomi A, Abualhasan M, Al-Ramahi R, Shraim N, et al. Formulation and bioequivalence of two Valsartan/Amlodipine immediate release tablets after a single oral administration. Pak J Pharm Sci. 2014;27(4):755-62.

10. Yacobi A, Masson E, Moros D, Ganes D, Lapointe C, Abolfathi Z, et al. Who needs individual bioequivalence studies for narrow therapeutic index drugs? A case for warfarin. J Clin Pharmacol. 2000;40(8):826-35.

11. Merali RM, Walker, S.E., Paton, T.W., Sheridan, B.L., Borst, S.I. Bioavailability and platelet function effect of acetylsalicylic acid. Canadian Journal of Clinical Pharmacology. 1996;3(1):29-33.

12. Lainesse A, Ozalp Y, Wong H, Alpan RS. Bioequivalence study of clopidogrel bisulfate film-coated tablets. Arzneimittelforschung. 2004;54(9A):600-4.

13. El Ahmady O, Ibrahim M, Hussein AM, Bustami RT. Bioequivalence of two oral formulations of clopidogrel tablets in healthy male volunteers. Int J Clin Pharmacol Ther. 2009;47(12):780-4.

14. Filipe A, Almeida S, Franco Spinola AC, Neves R, Tanguay M, Jimenez C, et al. Single-dose randomized, open-label, 2-way crossover bioequivalence study of clopidogrel 75 mg tablet in healthy volunteers under fasting conditions. Int J Clin Pharmacol Ther. 2009;47(3):187-94.

15. Richter W, Erenmemisoglu A, Van der Meer MJ, Emritte N, Tuncay E, Koytchev R. Bioequivalence study of two different clopidogrel bisulfate film-coated tablets. Arzneimittelforschung. 2009;59(6):297-302.

16. Khan SB, Hameedullah, Noor L, Hafeezullah M, Awan ZA, ud Din S. Comparison of effect of locally available brands of clopidogrel on platelet aggregation in patients with coronary artery disease. J Ayub Med Coll Abbottabad. 2010;22(1):115-7.

17. Setiawati E, Yunaidi DA, Handayani LR, Santoso ID, Setiawati A, Tjandrawinata RR. Bioequivalence study of two clopidogrel film-coated tablet formulations in healthy volunteers. Arzneimittelforschung. 2011;61(12):681-4.

18. Sambu N, Radhakrishnan A, Curzen N. A randomized crossover study comparing the antiplatelet effect of plavix versus generic clopidogrel. J Cardiovasc Pharmacol. 2012;60(6):495-501.

19. Eldon MA, Kinkel AW, Daniel JE, Latts JR. Bioavailability of propranolol hydrochloride tablet formulations: application of multiple dose crossover studies. Biopharm Drug Dispos. 1989;10(1):69-76.

20. Rostock G, Vogel I, Stulich M, Gunzel R. Bioequivalence of a new atenolol formulation. Int J Clin Pharmacol Ther Toxicol. 1992;30(11):482-3.

21. Yuen KH, Peh KK, Chan KL, Toh WT. Pharmacokinetic and bioequivalent study of a generic Metoprolol tablet preparation. Drug Dev Ind Pharm. 1998;24(10):955-9.

22. Peh KK, Yuen KH, Wong JW, Toh WT. Comparative bioavailability study of two atenolol tablet preparations. Drug Dev Ind Pharm. 1999;25(3):357-60.

23. Rojanasthien N, Manorot M, Kumsorn B. Bioequivalence study of generic atenolol tablets in healthy Thai volunteers. J Med Assoc Thai. 1999;82(9):907-14.

24. Jovanovic D, Cusic S, Rancic D, Srnic D, Perkovic-Vukcevic N. A pharmacokinetic comparison of generic tablets containing bisoprolol with the innovator formulation in healthy volunteers. J Clin Pharmacol. 2006;46(10):1217-22.

25. Carter BL, Noyes MA, Demmler RW. Differences in serum concentrations of and responses to generic verapamil in the elderly. Pharmacotherapy. 1993;13(4):359-68.

26. Waldman SA, Morganroth J. Effects of food on the bioequivalence of different verapamil sustained-release formulations. J Clin Pharmacol. 1995;35(2):163-9.

27. Tsang YC, Pop R, Gordon P, Hems J, Spino M. High variability in drug pharmacokinetics complicates determination of bioequivalence: experience with verapamil. Pharm Res. 1996;13(6):846-50.

28. Rojanasthien N, Teekachunhatean S, Jakob K, Gaupp M, Arnold P, Chaichana N, et al. Bioequivalence study of generic amlodipine in healthy Thai male volunteers. Int J Clin Pharmacol Ther. 2004;42(6):330-5.

29. Sailer R, Arnold P, Erenmemisoglu A, Martin W, Tamur U, Kanzik I, et al. Pharmacokinetics and bioequivalence study of a generic amlodipine tablet formulation in healthy male volunteers. Arzneimittelforschung. 2007;57(7):462-6.

30. Barbhaiya RH, Patel RB, Corrick-West HP, Joslin RS, Welling PG. Comparative bioavailability and pharmacokinetics of hydrochlorothiazide from oral tablet dosage forms, determined by plasma level and urinary excretion methods. Biopharm Drug Dispos. 1982;3(4):329-36.

31. Maitai CK, Ogeto JO, Munenge RW, Ochieng S, Juma FD. A comparative study of the efficacy of seven brands of frusemide tablets. East Afr Med J. 1984;61(1):6-10.

32. Shah VP, Walker MA, Prasad VK, Lin J, Knapp G, Cabana BE. Preliminary observations on dissolution and bioavailability of triamterene-hydrochlorothiazide combination products. Biopharm Drug Dispos. 1984;5(1):11-9.

33. Hamidi M, Shahbazi MA, Azimi K. Bioequivalence evaluation of a triamterene-hydrochlorothiazide generic product: a new bioequivalence index for fixed-dose combinations. Regul Toxicol Pharmacol. 2011;59(1):149-56.

34. Shaw TR, Raymond K, Howard MR, Hamer J. Therapeutic non-equivalence of digoxin tablets in the United Kingdom: correlation with tablet dissolution rate. Br Med J. 1973;4(5895):763-6.

35. Zinny MA, Taggart WV. Bioavailability of two manufacturers' sustained-release quinidine gluconate tablets at steady state. Clin Ther. 1984;7(1):22-7.

36. Hilleman DE, Patterson AJ, Mohiuddin SM, Ortmeier BG, Destache CJ. Comparative bioequivalence and efficacy of two sustained-release procainamide formulations in patients with cardiac arrhythmias. Drug Intell Clin Pharm. 1988;22(7-8):554-8.

37. Valecha N, Gupta U, Mehta VL. Comparative bioequivalence study of different brands of acetyl salicylic acid in human volunteers. Eur J Drug Metab Pharmacokinet. 1993;18(3):251-3.

38. Huang J, Chen R, Wei C, Li R, Yuan G, Liu X, et al. Pharmacokinetics and bioequivalence evaluation of two acipimox tablets: a single-dose, randomized-sequence, two-way crossover study in healthy Chinese male volunteers. Drug Res (Stuttg). 2013;63(2):79-83.

39. Najib NM, Idkaidek N, Adel A, Admour I, Astigarraga RE, Nucci GD, et al. Pharmacokinetics and bioequivalence evaluation of two simvastatin 40 mg tablets (Simvast and Zocor) in healthy human volunteers. Biopharm Drug Dispos. 2003;24(5):183-9.

40. Mulla H, Hussain N, Tanna S, Lawson G, Manktelow BN, Tuleu C, et al. Assessment of liquid captopril formulations used in children. Arch Dis Child. 2011;96(3):293-6.

41. Gasche D, Ulle T, Meier B, Greiner RA. Cost-effectiveness of ticagrelor and generic clopidogrel in patients with acute coronary syndrome in Switzerland. Swiss Med Wkly. 2013;143:w13851.

42. Theidel U, Asseburg C, Giannitsis E, Katus H. Cost-effectiveness of ticagrelor versus clopidogrel for the prevention of atherothrombotic events in adult patients with acute coronary syndrome in Germany. Clin Res Cardiol. 2013;102(6):447-58.

43. Faasse K, Cundy T, Gamble G, Petrie KJ. The effect of an apparent change to a branded or generic medication on drug effectiveness and side effects. Psychosom Med. 2013;75(1):90-6.

44. Brown MJ, Toal CB. Formulation of long-acting nifedipine tablets influences the heart rate and sympathetic nervous system response in hypertensive patients. Br J Clin Pharmacol. 2008;65(5):646-52.

45. Roca-Cusachs A, Aracil-Vilar J, Calvo-Gomez C, Vaquer-Perez JV, Laporta-Crespo F, Rojas-Serrano MJ, et al. Clinical effects of torasemide prolonged release in mild-to-moderate hypertension: a randomized noninferiority trial versus torasemide immediate release. Cardiovasc Ther. 2008;26(2):91-100.

46. Kasmer RJ, Nara AR, Green JA, Chawla AK, Fleming GM. Comparable steady-state bioavailability between two preparations of conventional-release procainamide hydrochloride. Drug Intell Clin Pharm. 1987;21(2):183-6.

47. Ghaffari S, Kazemi B, Golzari IG. Efficacy of a new accelerated streptokinase regime in acute myocardial infarction: a double blind randomized clinical trial. Cardiovasc Ther. 2013;31(1):53-9.

48. Arsenault KA, Paikin JS, Hirsh J, Dale B, Whitlock RP, Teoh K, et al. Subtle differences in commercial heparins can have serious consequences for cardiopulmonary bypass patients: A randomized controlled trial. J Thorac Cardiovasc Surg. 2012;144(4):944-50 e3.

49. McPherson R, Hanna K, Agro A, Braeken A. Cerivastatin versus branded pravastatin in the treatment of primary hypercholesterolemia in primary care practice in Canada: a one-year, open-label, randomized, comparative study of efficacy, safety, and cost-effectiveness. Clin Ther. 2001;23(9):1492-507.

50. Park S, Kang HJ, Rim SJ, Ha JW, Oh BH, Chung N, et al. A randomized, open-label study to evaluate the efficacy and safety of pitavastatin compared with simvastatin in Korean patients with hypercholesterolemia. Clin Ther. 2005;27(7):1074-82.

51. Rodriguez-Roa E, Tellez, R., Rodriguez, F., Gonzalez, M. Evaluación comparativa de la efectividad clínica de dos formulaciones de atorvastatina en pacientes con o sin enfermedad cardiovascular: Estudio Multicéntrico Nacional. Revista Latinoamericana de Hipertensión. 2007;3(4):129-35.

52. Tran YB, Frial T, Miller PS. Statin's cost-effectiveness: a Canadian analysis of commonly prescribed generic and brand name statins. Can J Clin Pharmacol. 2007;14(2):e205-14.

53. Ballantyne CM, Davidson MH, McKenney J, Keller LH, Bajorunas DR, Karas RH. Comparison of the safety and efficacy of a combination tablet of niacin extended release and simvastatin vs simvastatin monotherapy in patients with increased non-HDL cholesterol (from the SEACOAST I study). Am J Cardiol. 2008;101(10):1428-36.

54. Insull W, Jr., Basile JN, Vo AN, Jiang P, Thakkar R, Padley RJ. Efficacy and safety of combination therapy with niacin extended-release and simvastatin versus atorvastatin in patients with dyslipidemia: The SUPREME Study. J Clin Lipidol. 2009;3(2):109-18.

55. Rodríguez-Roa E, Téllez, R.,Rodríguez, F., González, M. Evaluacion comparativa de la efectividad clinica de dos formullaciones de atorvastatina: Atorvastatina amorfa de Laboratorios LETI (ATO-L) y Atorvastatina cristalina de Laboratorios PFIZER marca Lipitor(R) (ATO-P) en pacientes con o sin enfermidad cardiovascular. Estudio Multicentrico Nacional. Archivos Venezolanos de Farmacología y Terapéutica. 2010;29(3):3-6.

56. Portoles A, Terleira A, Almeida S, Garcia-Arenillas M, Caturla MC, Filipe A, et al. Bioequivalence study of two formulations of enalapril, at a single oral dose of 20 mg (tablets): A randomized, two-way, open-label, crossover study in healthy volunteers. Curr Ther Res Clin Exp. 2004;65(1):34-46.

57. Carranza MJ, Alvarado, J.M.N., Aguirre, C.I.A. Therapeutic equivalence of only dose of three presentations of captopril in women with essential hypertension. Medicina Interna de Mexico. 2005;21(4):273-81.

58. Kim SH, Chung WY, Zo JH, Kim MA, Chang HJ, Cho YS, et al. Efficacy and tolerability of two formulations of ramipril in Korean adults with mild to moderate essential hypertension: an 8-week, multicenter, prospective, randomized, open-label, parallel-group noninferiority trial. Clin Ther. 2009;31(5):988-98.

59. Spinola AC, Almeida S, Filipe A, Neves R, Trabelsi F, Farre A. Results of a single-center, single-dose, randomized-sequence, open-label, two-way crossover bioequivalence study of two formulations of valsartan 160-mg tablets in healthy volunteers under fasting conditions. Clin Ther. 2009;31(9):1992-2001.

60. Iqbal M, Khuroo A, Batolar LS, Tandon M, Monif T, Sharma PL. Pharmacokinetics and bioequivalence study of three oral formulations of valsartan 160 mg: a single-dose, randomized, open-label, three-period crossover comparison in healthy Indian male volunteers. Clin Ther. 2010;32(3):588-96.

61. Jia JY, Zhang MQ, Liu YM, Liu Y, Liu GY, Li SJ, et al. Pharmacokinetics and bioequivalence evaluation of two losartan potassium 50-mg tablets: A single-dose, randomized-sequence, open-label, two-way crossover study in healthy Chinese male volunteers. Clin Ther. 2010;32(7):1387-95.

62. Li KY, Liang JP, Hu BQ, Qiu Y, Luo CH, Jiang Y, et al. The relative bioavailability and fasting pharmacokinetics of three formulations of olmesartan medoxomil 20-mg capsules and tablets in healthy Chinese male volunteers: An open-label, randomized-sequence, single-dose, three-way crossover study. Clin Ther. 2010;32(9):1674-80.

63. Larouche R. NCT01124162 (Losartan 100 mg Tablets in Healthy Subjects Under Fasting Conditions). ClinicalTrialsgov. 2010.

64. Larouche R. NCT01124175 (Losartan 100 mg Tablet in Healthy Subjects Under Non-Fasting Conditions). ClinicalTrialsgov. 2010.

65. Carlson JD. NCT01149473 (Losartan Potassium/Hydrochlorothiazide 100/25 mg Tablets in Healthy Subjects Under Non-Fasting Conditions). ClinicalTrialsgov. 2010.

66. Carlson JD. NCT01149486 (Losartan Potassium/Hydrochlorothiazide 100/25 mg Tablets in Healthy Subjects Under Fasting Conditions). ClinicalTrialsgov. 2010.

67. Oigman W, Gomes MA, Pereira-Barretto AC, Povoa R, Kohlmann O, Rocha JC, et al. Efficacy and safety of two ramipril and hydrochlorothiazide fixed-dose combination formulations in adults with stage 1 or stage 2 arterial hypertension evaluated by using ABPM. Clin Ther. 2013;35(5):702-10.

68. Handler J, Nguyen, T.T., Rush, S., Pham, N.T. A blinded, randomized, crossover study comparing the efficacy and safety of generic Warfarin sodium to Coumadin. Preventive Cardiology. 1998;1(4):13-20.

69. Neutel JM, Smith, D.H. A randomized crossover study to compare the efficacy and tolerability of Barr warfarin sodium to the currently available Coumadin. Cardiovasc Rev Rep. 1998;19(2):49-59.

70. Weibert RT, Yeager BF, Wittkowsky AK, Bussey HI, Wilson DB, Godwin JE, et al. A randomized, crossover comparison of warfarin products in the treatment of chronic atrial fibrillation. Ann Pharmacother. 2000;34(9):981-8.

71. Lee HL, Kan CD, Yang YJ. Efficacy and tolerability of the switch from a branded to a generic warfarin sodium product: an observer-blinded, randomized, crossover study. Clin Ther. 2005;27(3):309-19.

72. Pereira JA, Holbrook AM, Dolovich L, Goldsmith C, Thabane L, Douketis JD, et al. Are brand-name and generic warfarin interchangeable? Multiple n-of-1 randomized, crossover trials. Ann Pharmacother. 2005;39(7-8):1188-93.

73. Rao TR, Usha PR, Naidu MU, Gogtay JA, Meena M. Bioequivalence and tolerability study of two brands of clopidogrel tablets, using inhibition of platelet aggregation and pharmacodynamic measures. Curr Ther Res Clin Exp. 2003;64(9):685-96.

74. Ashraf T, Ahmed M, Talpur MS, Kundi A, Faruqui AM, Jaffery AH, et al. Competency profile of locally manufactured clopidogrel Lowplat and foreign manufactured clopidogrel Plavix in patients of suspected ischemic heart disease (CLAP-IHD). J Pak Med Assoc. 2005;55(10):443-8.

75. Mijares M, Gomez M, Quijada A, Borges R, Ruiz-Saez A. Eficacia comparativa de dos presentaciones de clopidogrel en la inhibiciòn de la agregaciòn plaquetaria. Arch Venezo Farmacol Terap. 2008;27(1):88-91.

76. Kim SD, Kang W, Lee HW, Park DJ, Ahn JH, Kim MJ, et al. Bioequivalence and tolerability of two clopidogrel salt preparations, besylate and bisulfate: a randomized, open-label, crossover study in healthy Korean male subjects. Clin Ther. 2009;31(4):793-803.

77. Di Girolamo G, Czerniuk P, Bertuola R, Keller GA. Bioequivalence of two tablet formulations of clopidogrel in healthy Argentinian volunteers: a single-dose, randomized-sequence, open-label crossover study. Clin Ther. 2010;32(1):161-70.

78. Muller A, Octavio J, Gonzalez MY, Contreras J, Mendez G, Portillo M, et al. Clinical bioequivalence of a dose of clopidogrel Leti Cravid tablets 75 mg versus clopidogrel Sanofi Plavix tablets 75 mg administered on a daily dose for 7 days on healthy volunteers: a clinical trial. Am J Ther. 2010;17(3):351-6.

79. Shim CY, Park S, Song JW, Lee SH, Kim JS, Chung N. Comparison of effects of two different formulations of clopidogrel bisulfate tablets on platelet aggregation and bleeding time in healthy Korean volunteers: A single-dose, randomized, open-label, 1-week, two-period, phase IV crossover study. Clin Ther. 2010;32(9):1664-73.

80. Khosravi AR, Pourmoghadas M, Ostovan M, Mehr GK, Gharipour M, Zakeri H, et al. The impact of generic form of Clopidogrel on cardiovascular events in patients with coronary artery stent: results of the OPCES study. J Res Med Sci. 2011;16(5):640-50.

81. Suh JW, Seung KB, Gwak CH, Kim KS, Hong SJ, Park TH, et al. Comparison of antiplatelet effect and tolerability of clopidogrel resinate with clopidogrel bisulfate in patients with coronary heart disease (CHD) or CHD-equivalent risks: a phase IV, prospective, double-dummy, parallel-group, 4-week noninferiority trial. Clin Ther. 2011;33(8):1057-68.

82. Oberhansli M, Lehner C, Puricel S, Lehmann S, Togni M, Stauffer JC, et al. A randomized comparison of platelet reactivity in patients after treatment with various commercial clopidogrel preparations: the CLO-CLO trial. Arch Cardiovasc Dis. 2012;105(11):587-92.

83. Srimahachota S, Rojnuckarin P, Udayachalerm W, Buddhari W, Chaipromprasit J, Lertsuwunseri V, et al. Comparison of original and generic clopidogrel 600 mg loading dose in the patients who planned undergoing coronary angiography. Journal of the Medical Association of Thailand = Chotmaihet thangphaet. 2012;95(12):1495-500.

84. Tsoumani ME, Kalantzi KI, Dimitriou AA, Ntalas IV, Goudevenos IA, Tselepis AD. Antiplatelet efficacy of long-term treatment with clopidogrel besylate in patients with a history of acute coronary syndrome: comparison with clopidogrel hydrogen sulfate. Angiology. 2012;63(7):547-51.

85. Tsoumani ME, Kalantzi KI, Dimitriou AA, Ntalas IV, Goudevenos IA, Tselepis AD. Effect of clopidogrel besylate on platelet reactivity in patients with acute coronary syndromes. Comparison with clopidogrel hydrogen sulfate. Expert Opin Pharmacother. 2012;13(2):149-58.

86. Zou JJ, Tan, J., Fan, H. W., Chen, S.L. Bioequivalence study of clopidogrel 75 mg tablets in healthy male volunteers. J Bioequiv Bioavalab. 2012;4:6-9.

87. Park JB, Koo BK, Choi WG, Kim SY, Park J, Kwan J, et al. Comparison of antiplatelet efficacy and tolerability of clopidogrel napadisilate with clopidogrel bisulfate in coronary artery disease patients after percutaneous coronary intervention: a prospective, multicenter, randomized, open-label, phase IV, noninferiority trial. Clin Ther. 2013;35(1):28-37 e4.

88. Komosa A, Siller-Matula JM, Kowal J, Lesiak M, Siniawski A, Maczynski M, et al. Comparison of the antiplatelet effect of two clopidogrel bisulfate formulations: Plavix and generic-Egitromb. Platelets. 2014.

89. Seo KW, Tahk SJ, Yang HM, Yoon MH, Shin JH, Choi SY, et al. Point-of-care Measurements of Platelet Inhibition After Clopidogrel Loading in Patients With Acute Coronary Syndrome: Comparison of Generic and Branded Clopidogrel Bisulfate. Clin Ther. 2014;36(11):1588-94.

90. Biswas NR, Garg SK, Kumar N, Mukherjee S, Sharma PL. Comparative pharmacokinetic and pharmacodynamic study of four different brands of propranolol in normal volunteers. Int J Clin Pharmacol Ther Toxicol. 1989;27(10):515-9.

91. Carter BL, Gersema LM, Williams GO, Schabold K. Once-daily propranolol for hypertension: a comparison of regular-release, long-acting, and generic formulations. Pharmacotherapy. 1989;9(1):17-22.

92. el-Sayed MS, Davies B. Effect of two formulations of a beta blocker on fibrinolytic response to maximum exercise. Med Sci Sports Exerc. 1989;21(4):369-73.

93. Chiang HT, Hou ZY, Lee DK, Wu TL, Chen CY. A comparison of antihypertensive effects between two formulations of atenolol: tenolol and tenormin. Zhonghua Yi Xue Za Zhi (Taipei). 1995;55(5):366-70.

94. Sarkar MA, Noonan, P.K., Adams, M.J., O’Donnell, J.P. Pharmacodynamic and pharmacokinetic comparisons to evaluate bioequivalence of atenolol. Clin Res Regul Aff. 1995;12(1):47-62.

95. Bongers V, Sabin, G.V. Comparison of the effect of two metoprolol formulations on total ischaemic burden. Clin Drug Invest. 1999;17:103-10.

96. Cuadrado A, Rodriguez Gascon A, Hernandez RM, Castilla AM, de la Maza A, Lopez de Ocariz A, et al. In vitro and in vivo equivalence of two oral atenolol tablet formulations. Arzneimittelforschung. 2002;52(5):371-8.

97. Mirfazaelian A, Tabatabaeifar, M, Rezaee, S, Mahmoudian, M. Bioequivalence study of atenolol. Daru J Faculty Pharm. 2003;11(3):95-8.

98. Portoles A, Filipe A, Almeida S, Terleira A, Vallee F, Vargas E. Bioequivalence study of two different tablet formulations of carvedilol in healthy volunteers. Arzneimittelforschung. 2005;55(4):212-7.

99. Bus-Kwasnik K, Ksycinska H, Les A, Serafin-Byczak K, Rudzki PJ, Raszek J, et al. Bioequivalence and pharmacokinetics of two 10-mg bisoprolol formulations as film-coated tablets in healthy white volunteers: a randomized, crossover, open-label, 2-period, single-dose, fasting study. Int J Clin Pharmacol Ther. 2012;50(12):909-19.

100. Liu Y, Lu C, Chen Q, Wang W, Liu GY, Lu XP, et al. Bioequivalence and pharmacokinetic evaluation of two tablet formulations of carvedilol 25-mg: a single-dose, randomized-sequence, open-label, two-way crossover study in healthy Chinese male volunteers. Drug Res (Stuttg). 2013;63(2):74-8.

101. Saseen JJ, Porter JA, Barnette DJ, Bauman JL, Zajac EJ, Jr., Carter BL. Postabsorption concentration peaks with brand-name and generic verapamil: a double-blind, crossover study in elderly hypertensive patients. J Clin Pharmacol. 1997;37(6):526-34.

102. Usha PR, Naidu, M.U.R., Kumar, T.R., Shobha, J.C., Vijay, T. Bioequivalence study of two slow-release diltiazem formulations using dynamic measures in healthy volunteers. Clin Drug Investig. 1997;14(6):482-86.

103. Park JY, Kim KA, Lee GS, Park PW, Kim SL, Lee YS, et al. Randomized, open-label, two-period crossover comparison of the pharmacokinetic and pharmacodynamic properties of two amlodipine formulations in healthy adult male Korean subjects. Clin Ther. 2004;26(5):715-23.

104. Kim SH, Kim YD, Lim DS, Yoon MH, Ahn YK, On YK, et al. Results of a phase III, 8-week, multicenter, prospective, randomized, double-blind, parallel-group clinical trial to assess the effects of amlodipine camsylate versus amlodipine besylate in Korean adults with mild to moderate hypertension. Clin Ther. 2007;29(9):1924-36.

105. Mignini F, Tomassoni D, Traini E, Amenta F. Single-dose, randomized, crossover bioequivalence study of amlodipine maleate versus amlodipine besylate in healthy volunteers. Clin Exp Hypertens. 2007;29(8):539-52.

106. Kim SA, Park S, Chung N, Lim DS, Yang JY, Oh BH, et al. Efficacy and safety profiles of a new S(-)-amlodipine nicotinate formulation versus racemic amlodipine besylate in adult Korean patients with mild to moderate hypertension: an 8-week, multicenter, randomized, double-blind, double-dummy, parallel-group, phase III, noninferiority clinical trial. Clin Ther. 2008;30(5):845-57.

107. Liu Y, Jia J, Liu G, Li S, Lu C, Yu C. Pharmacokinetics and bioequivalence evaluation of two formulations of 10-mg amlodipine besylate: an open-label, single-dose, randomized, two-way crossover study in healthy Chinese male volunteers. Clin Ther. 2009;31(4):777-83.

108. Garg SK, Gupta U, Mathur VS. Comparative bioequivalence study of furosemide in human volunteers. Int J Clin Pharmacol Ther Toxicol. 1984;22(11):618-20.

109. Grahnen A, Hammarlund M, Lundqvist T. Implications of intraindividual variability in bioavailability studies of furosemide. Eur J Clin Pharmacol. 1984;27(5):595-602.

110. Martin BK, Uihlein M, Ings RM, Stevens LA, McEwen J. Comparative bioavailability of two furosemide formulations in humans. J Pharm Sci. 1984;73(4):437-41.

111. Pan HY, Wang RY, Chan TK. Efficacy of two proprietary preparations of frusemide in patients with congestive heart failure. Med J Aust. 1984;140(4):221-2.

112. Meyer BH, Muller FO, Swart KJ, Luus HG, Werkman IM. Comparative bio-availability of four formulations of furosemide. S Afr Med J. 1985;68(9):645-7.

113. Singh A, Gupta U, Sagar S, Garg SK, Sharma BK, Mathur VS. Comparative bioequivalence study of furosemide in patients with edema of renal origin. Int J Clin Pharmacol Ther Toxicol. 1987;25(3):136-8.

114. Sharoky M, Perkal M, Tabatznik B, Cane RC, Jr., Costello K, Goodwin P. Comparative efficacy and bioequivalence of a brand-name and a generic triamterene-hydrochlorothiazide combination product. Clin Pharm. 1989;8(7):496-500.

115. Kaojarern S, Poobrasert O, Utiswannakul A, Kositchaiwat U. Bioavailability and pharmacokinetics of furosemide marketed in Thailand. J Med Assoc Thai. 1990;73(4):191-7.

116. Awad R, Arafat T, Saket M, Saleh M, Gharaibeh M, Zmeili S, et al. A bioequivalence study of two products of furosemide tablets. Int J Clin Pharmacol Ther Toxicol. 1992;30(1):18-23.

117. Murray MD, Haag KM, Black PK, Hall SD, Brater DC. Variable furosemide absorption and poor predictability of response in elderly patients. Pharmacotherapy. 1997;17(1):98-106.

118. Almeida S, Pedroso P, Filipe A, Pinho C, Neves R, Jimenez C, et al. Study on the bioequivalence of two formulations of eplerenone in healthy volunteers under fasting conditions: data from a single-center, randomized, single-dose, open-label, 2-way crossover bioequivalence study. Arzneimittelforschung. 2011;61(3):153-9.

119. Kumar S, Monif T, Khuroo A, Reyar S, Jain R, Singla AK, et al. Pharmacokinetic comparison and bioequivalence evaluation of losartan/ hydrochlorothiazide tablet between Asian Indian and Japanese volunteers. Int J Clin Pharmacol Ther. 2014;52(1):39-54.

120. Assawawitoontip S, Wiwanitkit V. A randomized crossover study to evaluate LDL-cholesterol lowering effect of a generic product of simvastatin (Unison Company) compared to simvastatin (Zocor) in hypercholesterolemic subjects. J Med Assoc Thai. 2002;85 Suppl 1:S118-24.

121. Wiwanitkit V, Wangsaturaka D, Tangphao O. LDL-cholesterol lowering effect of a generic product of simvastatin compared to simvastatin (Zocor) in Thai hypercholesterolemic subjects -- a randomized crossover study, the first report from Thailand. BMC Clin Pharmacol. 2002;2:1.

122. Kim SH, Park K, Hong SJ, Cho YS, Sung JD, Moon GW, et al. Efficacy and tolerability of a generic and a branded formulation of atorvastatin 20 mg/d in hypercholesterolemic Korean adults at high risk for cardiovascular disease: a multicenter, prospective, randomized, double-blind, double-dummy clinical trial. Clin Ther. 2010;32(11):1896-905.

123. Liu YM, Pu HH, Liu GY, Jia JY, Weng LP, Xu RJ, et al. Pharmacokinetics and bioequivalence evaluation of two different atorvastatin calcium 10-mg tablets: A single-dose, randomized-sequence, open-label, two-period crossover study in healthy fasted Chinese adult males. Clin Ther. 2010;32(7):1396-407.

124. Boh M, Opolski G, Poredos P, Ceska R, Jezovnik M. Therapeutic equivalence of the generic and the reference atorvastatin in patients with increased coronary risk. Int Angiol. 2011;30(4):366-74.

125. Kim SH, Seo MK, Yoon MH, Choi DH, Hong TJ, Kim HS. Assessment of the efficacy and tolerability of 2 formulations of atorvastatin in Korean adults with hypercholesterolemia: a multicenter, prospective, open-label, randomized trial. Clin Ther. 2013;35(1):77-86.

126. Tsai YS, Lan SK, Ou JH, Tzai TS. Effects of branded versus generic terazosin hydrochloride in adults with benign prostatic hyperplasia: a randomized, open-label, crossover study in Taiwan. Clin Ther. 2007;29(4):670-82.

127. Feng L, Shen-Tu J, Liu J, Chen J, Wu L, Huang M. Bioequivalence of generic and branded subcutaneous enoxaparin: a single-dose, randomized-sequence, open-label, two-period crossover study in healthy Chinese male subjects. Clin Ther. 2009;31(7):1559-67.

128. Palmer JL, Kunhihitlu, A., Costantini, A., Esquivel, F., Roush, J., Edwards, K., Hill, T.W.K. Pharmacokinetic bioequivalence crossover study of branded generic and innovator formulations of the cholesterol lowering agent ezetimibe. Clinical Pharmacology in Drug Development. 2014;3(3):242-8.
