# Supplementary material for: Generic versus brand-name drugs used in cardiovascular diseases
Source: Eur J Epidemiol. 2015 Nov 30;31:351–68. doi: 10.1007/s10654-015-0104-8 (PMC4877434; doi:10.1007/s10654-015-0104-8)
Supplement: Supplementary file 2 — Supplementary material 2 (DOC 60 kb) [file 10654_2015_104_MOESM2_ESM.doc]

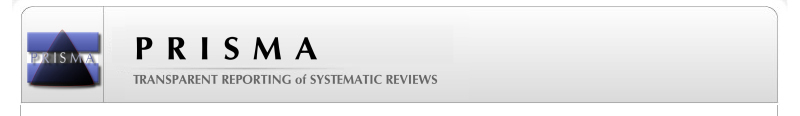
**PRISMA 2009 Flow Diagram**

**Screening**

**Included**

**Eligibility**

**Identification**

Records identified through database searching
(n=713*, including 123 protocols posted in ClinicalTrials.gov)

Additional records identified through other sources
(n=32)

Records after duplicates removed
(n=568)

Records screened
(n=568)

Records excluded
(n=433)

Full-text articles assessed for eligibility
(n =135)

Full-text articles excluded, with reasons
(n=61)

- 38 trials with bioequivalence data only

- 6 trials compared different formulations of the same drug

- 8 trials compared different drugs

- 1 trial used a placebo drug

- 1 trial data could not be extracted

Studies included in qualitative synthesis
(n=74)

Studies included in quantitative synthesis (meta-analysis)
(n=74)

- 70 published trials

- 4 trials with results posted in ClinicalTrials.gov
